# Molecular Diversity of Midbrain Development in Mouse, Human, and Stem Cells

**DOI:** 10.1016/j.cell.2016.09.027

**Published:** 2016-10-06

**Authors:** Gioele La Manno, Daniel Gyllborg, Simone Codeluppi, Kaneyasu Nishimura, Carmen Salto, Amit Zeisel, Lars E. Borm, Simon R.W. Stott, Enrique M. Toledo, J. Carlos Villaescusa, Peter Lönnerberg, Jesper Ryge, Roger A. Barker, Ernest Arenas, Sten Linnarsson

**Affiliations:** 1 Laboratory of Molecular Neurobiology, Department of Medical Biochemistry and Biophysics, Karolinska Institutet, 17177 Stockholm, Sweden; 2 Science for Life Laboratory, 17121 Solna, Sweden; 3 Department of Physiology and Pharmacology, Karolinska Institutet, 17177 Stockholm, Sweden; 4 John van Geest Centre for Brain Repair, Department of Clinical Neurosciences, University of Cambridge, Cambridge CB2 0PY, UK; 5 Psychiatric Stem Cell Group, Neurogenetics Unit, Center for Molecular Medicine, Karolinska University Hospital, 17176 Stockholm, Sweden; 6 Laboratory of Neural Microcircuitry, Brain Mind Institute, Ecole Polytechnique Federale de Lausanne, CH-1015 Lausanne, Switzerland

**Keywords:** dopaminergic neuron, ventral midbrain, human, mouse, single-cell RNA-seq

## Abstract

Understanding human embryonic ventral midbrain is of major interest for Parkinson’s disease. However, the cell types, their gene expression dynamics, and their relationship to commonly used rodent models remain to be defined. We performed single-cell RNA sequencing to examine ventral midbrain development in human and mouse. We found 25 molecularly defined human cell types, including five subtypes of radial glia-like cells and four progenitors. In the mouse, two mature fetal dopaminergic neuron subtypes diversified into five adult classes during postnatal development. Cell types and gene expression were generally conserved across species, but with clear differences in cell proliferation, developmental timing, and dopaminergic neuron development. Additionally, we developed a method to quantitatively assess the fidelity of dopaminergic neurons derived from human pluripotent stem cells, at a single-cell level. Thus, our study provides insight into the molecular programs controlling human midbrain development and provides a foundation for the development of cell replacement therapies.

## Highlights

• Species differences in developmental timing and cell proliferation
• Multiple radial glia subtypes biased toward distinct fates
• Adult dopaminergic neuron subtypes emerge postnatally
• A machine learning method to score dopaminergic differentiation of stem cells

## Graphical Abstract

**Figure:**
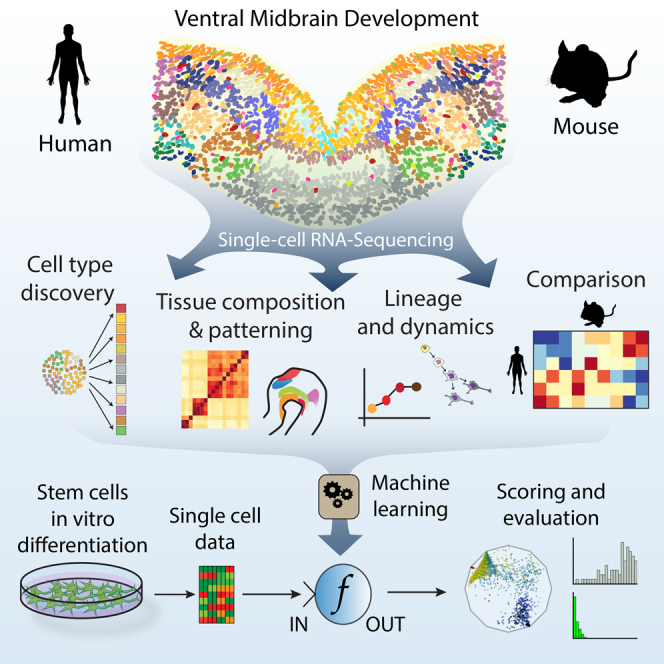

## Teaser

Analyzing the time course of ventral midbrain development in mouse, human, and stem cells by single-cell RNA-sequencing provides insight into dopaminergic neuron development and offers a strategy to assess the composition of stem-cell-derived preparations for clinical applications.

## Introduction

Much of our current knowledge about brain development is based on the rodent brain. In the embryonic mouse ventricular zone (VZ), neuroepithelial stem cells differentiate into radial glia that will generate neurons, astrocytes, oligodendrocytes, and ependymal cells in successive waves of differentiation (Shen et al., 2006). Analysis of mutant mice has revealed that morphogens such as WNT/β-catenin, SHH, and FGF8 induce regional-specific transcription factors at the midbrain-hindbrain boundary that provide nascent neuroblasts with defined dorso-ventral, antero-posterior, and mediolateral identities (Arenas et al., 2015). As a result, these neuroblasts mature into spatially defined mature populations, including dopaminergic neurons, oculomotor and trochlear neurons, and red nucleus neurons. The adult midbrain contains two main anatomically defined populations of dopaminergic neurons, located in the ventral tegmental area (VTA) and in the substantia nigra pars compacta (SNc) (Björklund and Dunnett, 2007). Notably, SNc neurons degenerate in Parkinson’s disease while the VTA suffers only a 40% reduction (Damier et al., 1999, Javoy-Agid and Agid, 1980).

The development of the human ventral midbrain is currently thought to follow a similar sequence of events and principles as in rodent. However, the cell type composition and developmental programs that control the human ventral midbrain are largely unknown. It is also unclear what is the degree of conservation between mouse and human midbrain development and whether all cell types in human even have unambiguous counterparts in the mouse. In addition, several fundamental questions remain to be elucidated. First, it is unclear whether a single cell type (radial glia; Bonilla et al., 2008) can give rise to all the diverse progeny found in the ventral midbrain. Second, although five molecularly distinct dopaminergic neuron types have been recently described in the adult mouse (Poulin et al., 2014), it is unclear if these are specified in the embryo (e.g., using patterning morphogens) or if they emerge only postnatally (e.g., as a result of local environmental cues or feedback from innervation targets).

Single-cell RNA-sequencing (RNA-seq) has been previously used for de novo cell type discovery in multiple tissues (Pollen et al., 2014, Pollen et al., 2015, Treutlein et al., 2014, Zeisel et al., 2015, Marques et al., 2016). Here, we use single-cell RNA-seq to examine ventral midbrain development in both mouse and human. Our results provide an unbiased classification of cell types and their gene expression patterns during human and mouse ventral midbrain development.

## Results and Discussion

### Development of the Mouse and Human Ventral Midbrain

**Figure 1:**
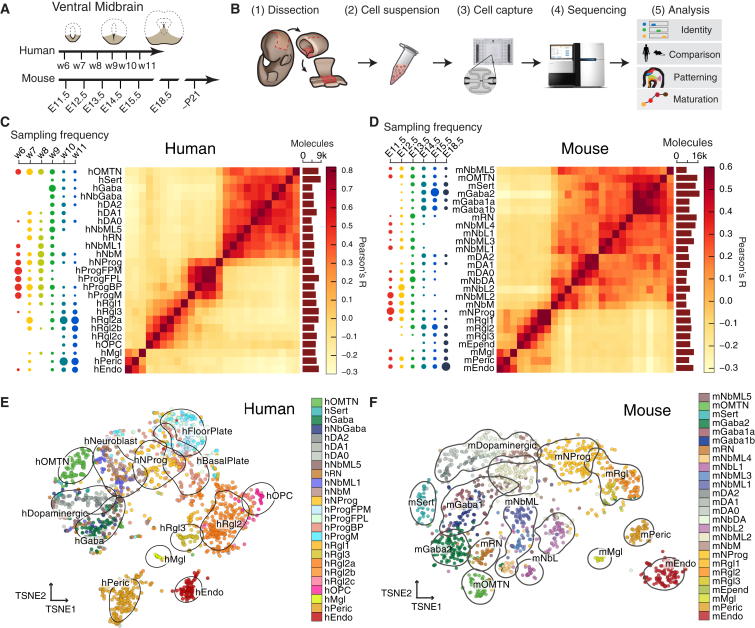
Cell Populations and their Distribution Over Time during Human and Mouse Ventral Midbrain Development (A) Overview of the time points sampled for human and mouse embryos. E, embryonic day; P, postnatal day; w, week. (B) Illustration of the workflow of the experiment and the region dissected. (C) Molecularly defined cell types of the human embryonic midbrain. Dot plot shows time distribution of cell types, heatmap shows pairwise correlations, and bars show average number of detected mRNA molecules per cell. Cell types are named using anatomical and functional mnemonics prefixed by “m” or “h” to indicate mouse and human respectively: OMTN, oculomotor and trochlear nucleus; Sert, serotonergic; NbM, medial neuroblast; NbDA, neuroblast dopaminergic; DA0-2, dopaminergic neurons; RN, red nucleus; Gaba1-2, GABAergic neurons; mNbL1-2, lateral neuroblasts; NbML1-5, mediolateral neuroblasts; NProg, neuronal progenitor; Prog, progenitor medial floorplate (FPM), lateral floorplate (FPL), midline (M), basal plate (BP); Rgl1-3, radial glia-like cells; Mgl, microglia; Endo, endothelial cells; Peric, pericytes; Epend, ependymal; OPC, oligodendrocyte precursor cells. (D) Molecularly defined cell types of the mouse embryonic midbrain. Cell types are named as above (C). (E) Human ventral midbrain single-cell transcriptomes visualized with t-Distributed stochastic neighbor embedding (t-SNE), colored by the clusters defined in (C). Contours are drawn to contain at least 80% of the cells belonging to the category. (F) Mouse ventral midbrain single-cell transcriptomes visualized with t-SNE, colored by the clusters defined in (D). Contours are drawn to contain at least 80% of the cells belonging to the category.

**Figure S1:**
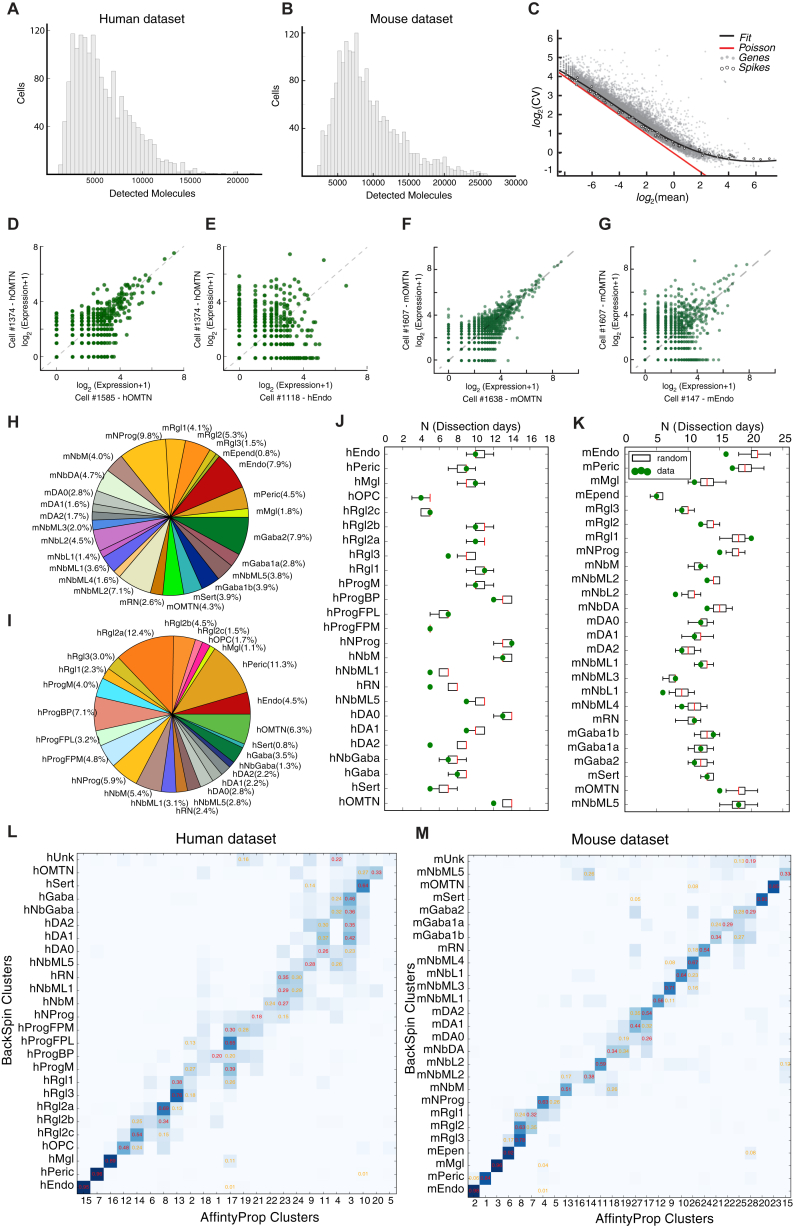
Quality Control of Single-Cell Rna-Seq, Related to Figure 1 (A) Distribution of number of mRNA molecules detected in human cells. (B) Distribution of number of mRNA molecules detected in mouse cells. (C) Plot of CV (coefficient of variation, i.e., SD divided by the mean) versus mean mRNA molecule counts. Grey dots, genes; red line, Poisson distribution; black curve, fit of noise distribution used to select genes with greater than expected CV. (D) Scatterplot showing genes expressed in two human cells of the same type. (E) Scatterplot showing genes expressed in two human cells of different types. (F) Scatterplot showing genes expressed in two mouse cells of the same type. (G) Scatterplot showing genes expressed in two mouse cells of different types. (H) Pie chart showing the cell type composition of mouse cell types, all time points combined (excluding adult). (I) Pie chart of human cell types as in (H). (J) Replicate experiments supporting each cell type in mouse. Box and whiskers plots showing the expected distribution of a perfectly random sampling procedure, estimated by repeatedly scrambling the gene labels. (box Q1-Q4; whiskers: 95% C.I.). Green dots show actual sampled data. (K) Replicate experiments supporting each cell type in human. Box and whiskers as in (J) (L) Heatmap showing the overlap of BackSPIN and Affinity Propagation clusters for the human dataset. (M) Heatmap showing the overlap of BackSPIN and Affinity Propagation clusters for the mouse dataset.

We performed unbiased single-cell RNA-seq at different developmental stages (Figures 1A, 1B, and S1A–S1G) covering dopaminergic progenitor specification, neurogenesis, and differentiation in human (Almqvist et al., 1996, Freeman et al., 1991). A total of 1,977 ventral midbrain cells were analyzed from ten human embryos (6–11 weeks; Table S1). In addition, we analyzed 1,907 single-cell transcriptomes from the mouse ventral midbrain, using a total of 271 embryos from 22 pooled litters covering six developmental time points (E11.5–E18.5; Table S1). 245 postnatal murine cells (77 *Th*^+^ neurons and 168 putative dopaminergic neurons sorted from *Slc6a3-Cre/tdTomato* mice by FACS [fluorescence-activated cell sorting]) were also examined.

Both mouse and human datasets were then analyzed in parallel using the same algorithms. We clustered the data using BackSPIN (Zeisel et al., 2015), resulting in a total of 25 (human) and 26 (mouse) clusters (Figures 1C–1F, S1H, and S1I). Similar results were obtained using affinity propagation (Figures S1L and S1M). Each cluster was supported by at least five independent litters (mouse) and four fetuses (human), and the number of animals contributing to each cluster matched expectations of random sampling (Figures S1J and S1K).

We combined RNA-seq markers, in situ hybridization, the time of sampling, and prior knowledge to name every cell transcriptional state that we found. Below, we use shorthand labels to indicate these clusters, prefixed to indicate the species (e.g., mRgl1 versus hRgl1 [mouse versus human radial glia-like cells type 1]). Using the embryo age as a variable, we tracked the appearance and disappearance of cell types during ventral midbrain development (Figures 1C and 1D).

**Figure S2:**
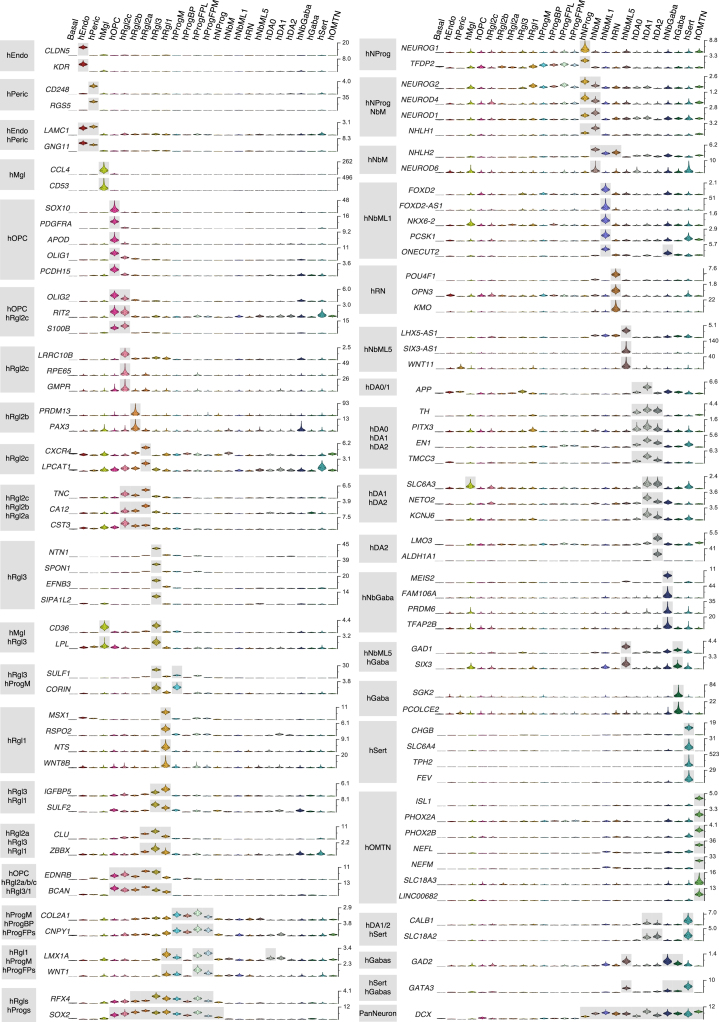
Violin Plots Showing a Selection of Genes with Specific Expression in Specific Human Cell Types, Related to Figure 1 Each row shows violin plots depicting posterior probability distributions for the expected mean expression, one for each cell type. Grey boxes indicate > 99.8% probability of expression above baseline (STAR Methods). Genes are grouped for clarity. A full set of genes is provided in Table S2.

The quantitative nature of the data (Figures S1A–S1G) allowed us to estimate the absolute level of expression for each gene in every cell type, in units of detected mRNA molecules per cell (with a detection efficiency of ∼20%; Zeisel et al., 2015), and to estimate the underlying cell-type-specific expression levels using a Bayesian generalized linear model (STAR Methods). A selection of genes that can be used to identify cell types is shown in Figures S2 (human) and S3 (mouse), and cell-type-specific transcriptional factor combinations for both human and mouse cells are shown in Figures S4A and S4B, respectively. The full set of differentially expressed genes is given in Table S2.

### Timing and Cell Proliferation Differ in Mouse and Human Development

Although embryos are of similar size at very early time points, the human brain outgrows the mouse by 1,000-fold (Herculano-Houzel, 2009). While most of the difference occurs in the forebrain, the human midbrain is also significantly larger than the murine (i.e., 300,000 hDA neurons versus 30,000 mDA [Nelson et al., 1996]). The difference is partly achieved by a longer gestation. However, a 10-fold increase in neuronal numbers would require only four extra cell divisions; thus, the timing and dynamics of human versus mouse midbrain development cannot be a simple matter of linear scaling.

**Figure 2:**
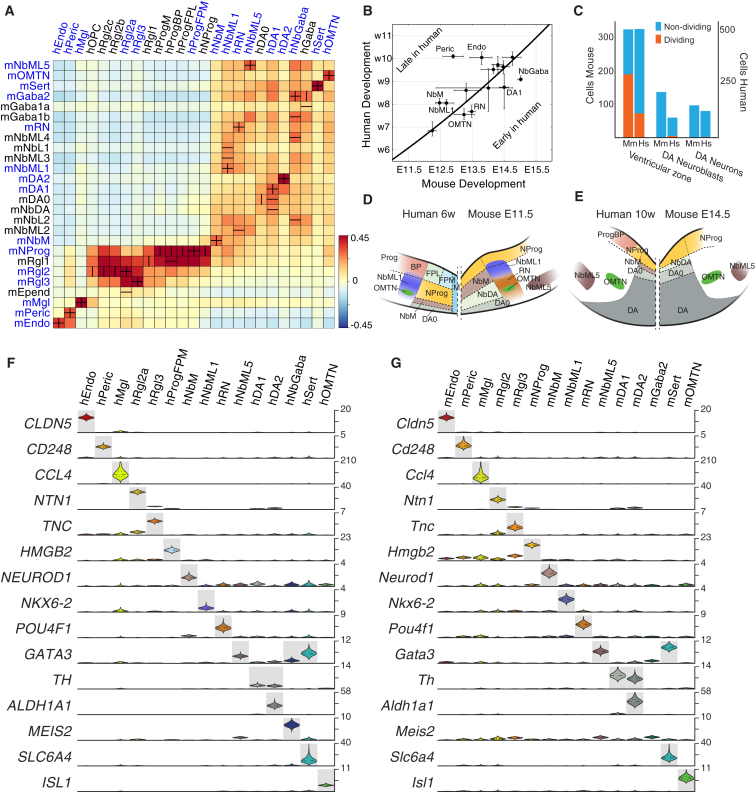
Human and Mouse Cell Type Homologies (A) Cross species similarity of all cell types in human (horizontally) and mouse (vertically). Vertical lines indicate highest correlation of human cell type to mouse. Horizontal lines indicate highest correlation of mouse cell type to human. Crosses are formed for cell types that are mutual best matches, indicated by blue text on cell type labels. (B) Time-course comparison of human versus mouse development, showing for each cell type the time point when half of all cells of that type have appeared. Solid curve shows the translation of mouse to human developmental time, based on the timing of key neurodevelopmental events. Error bars show 95% confidence intervals. (C) Number of cells representing stages of the dopaminergic lineage in the mouse and human. Cells were assigned to VZ (mRgl1-3, mNProg, and mEpend), dopaminergic neuroblasts (mNbM, mNbDA), and dopaminergic neurons (mDA0-2). Putatively dividing cells, as determined by the proliferation index, are shown in orange. (D) Schematic of early human and mouse cell-type compartments (not to scale). (E) Schematic of late human and mouse cell-type compartments (not to scale). (F) Human genes specifically expressed in mutually best-matching cell types in (A), shown as violin plots. Grey, enriched over baseline with posterior probability >99.8%. Right axis shows absolute molecule counts. (G) Same genes as in (F) but for the corresponding mouse cell types, shown as violin plots. Grey, enriched over baseline with posterior probability >99.8%. Right axis shows absolute molecular counts. Note that the vertical scales are the same as in (F).

In order to shed light on this process, we aligned human cell types with their mouse counterparts using a pairwise correlation of homologous genes (Figure 2A). Non-neural cell types (Endo [endothelial]; Peric [pericytes]; Mgl [microglia]) were mutual best matches, as were some of the mature neuronal cell types (OMTN, [oculomotor and trochlear nucleus]; RN [red nucleus]; DA0-2 [dopaminergic 0-2]; Sert [serotonergic]). Several neuroblast types were also mutual best matches, including medial neuroblasts (NbM), the precursors of dopaminergic neurons. However, two immature dopaminergic cell types in the mouse (mNbDA and mDA0) corresponded to only a single type in the human (hDA0). In addition, radial glia-like cells Rgl2 and Rgl3 matched, whereas earlier mRgl1 and mouse neuronal progenitor (mNProg) had a more complex relationship with human progenitor cell types due to changes over time, as discussed below.

Plotting the time of appearance of each cell type (relative to a previously published multispecies model based on key neurodevelopmental events, Workman et al., 2013; STAR Methods), we found that several neuronal types appeared at homologous time points (Figure 2B). However, Peric and Endo, as well as NbM and mediolateral neuroblasts (NbML), were found later in the human, whereas GABAergic neuroblasts, DA1, OMTN, and RN appeared early. Thus, specific intermediate cell types follow different timelines from one species to another, in which for instance, the transition from NbML to DA1 was relatively shorter in human than in mouse.

We then examined the proportion of VZ cells, intermediate cells in the dopaminergic lineage (NbM, NbDA and DA0), and dopaminergic neurons (DA1 and DA2) in the two species during development. We found a relative depletion of human neuroblasts (Figures 2C–2E) compared to the mouse. We hypothesized that these differences were the result of human progenitors dividing less frequently (although the total number of cell divisions would be larger; Pollen et al., 2015), such that at any given time point there would be fewer cells differentiating from progenitor cells to neurons. We computed a proliferation index based on an unsupervised selection of cell-cycle-associated genes (STAR Methods), classifying each cell as proliferative or not. In agreement with our hypothesis, human VZ cells in the dopaminergic lineage were only about half as proliferative as those of the mouse (Figure 2C). Thus, the larger human ventral midbrain is generated by less proliferative progenitors active over a longer period of time.

Finally, we examined the extent to which the expression levels of cell-type-specific genes are conserved between species, exploiting the fact that we had measured absolute mRNA molecule counts. We collected all homologous genes that were enriched (independently in each species) in homologous cell types and plotted their expression (Figures 2F and 2G). As a control, we examined the maximal expression of all genes in the cell types. Cell-type-specific, absolute gene expression levels were strikingly conserved (Figure S6H), with an overall correlation coefficient of *r*^2^ = 0.5, which was significantly greater than the correlation of non-cell-type-specific gene expression (*r*^2^ = 0.28, p < 2 × 10^−12^). Hence, genes expressed in homologous cell types tend to have retained their specific absolute expression levels, suggesting that they perform a similar function. In contrast, genes that have diverged such that they are no longer expressed in homologous cell types have also diverged in expression levels, suggesting that they have been co-opted to perform distinct functions or that they now perform the same function in distinct environments requiring different expression levels.

### A Diversity of Radial Glial Cell Types

**Figure S3:**
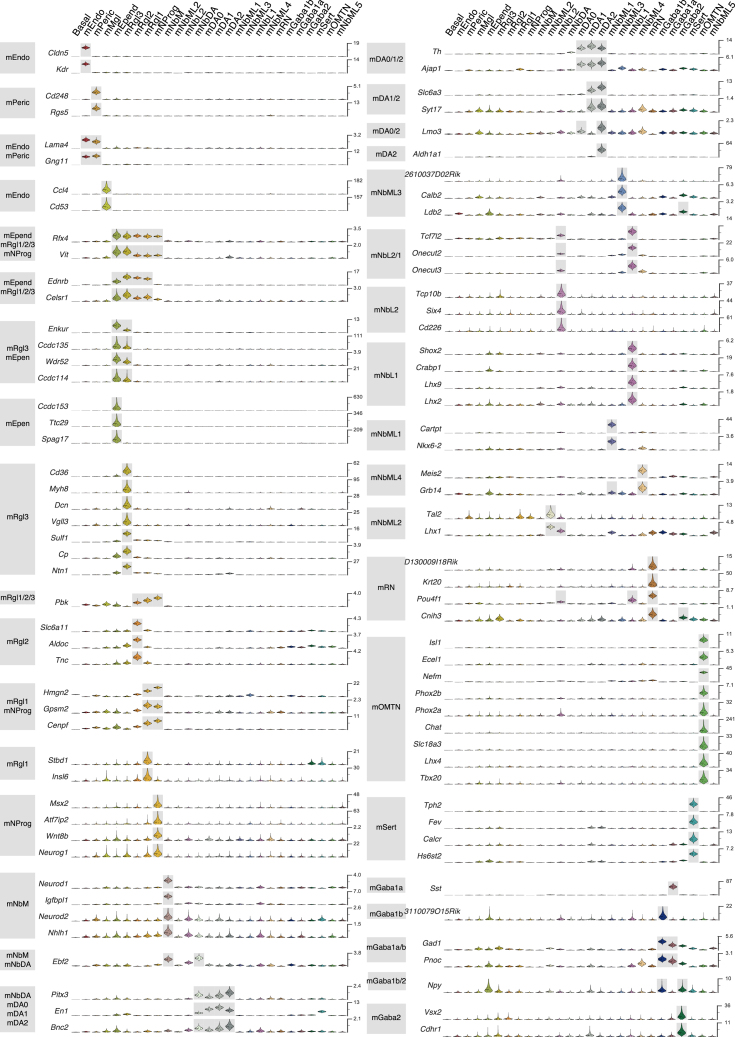
Violin Plots Showing a Selection of Genes with Specific Expression in Specific Mouse Cell Types, Related to Figure 1 As in Figure S2.

**Figure S4:**
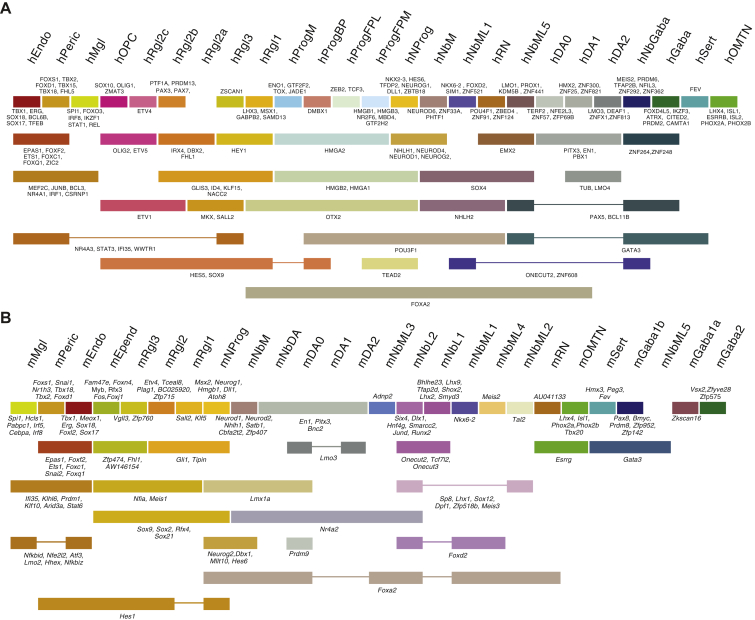
Transcription Factor Expression across Mouse and Human Cell Types, Related to Figure 1 (A) The binarized expression of transcription factors in human embryos. Rectangles are drawn below single or multiple cell types to represent binary patterns of expression. For each pattern, the names of the transcription factor genes expressed above baseline levels are indicated. (B) The binarized expression of transcription factors in mouse embryo.

We found five distinct cell types in the mouse VZ: a progenitor (mNProg), three distinct radial glia-like cell types (mRgl1–3), and ependymal cells (mEpend), which all shared the expression of the transcription factors *Sox2*, *Sox9*, *Sox21*, *and Rfx4* (Figures S3 and S4B). In human, the diversity was even greater, with eleven cell types: five early progenitor cells, five radial glia-like cell types (hRgl1, hRgl2a, hRgl2b, hRgl2c, hRgl3), and oligodendrocyte precursor cells (OPCs). All of these cells shared the expression of *SOX2*, while *RFX4* was not observed in hNProg, hRgl2c, and hOPC (Figures S2 and S4A). All hRgl and hOPCs expressed *SOX9* and *HES5*, and each of them was also unambiguously identified by the expression of individual or combinations of transcription factors (Figure S4A). Comparison between the two species revealed the exclusive expression of *Sox2* in all VZ cell types and that all radial glia-like cells share *Fabp7*, coding for a fatty acid binding protein induced by Notch signaling (Anthony et al., 2005). However, differences in transcription factor expression such as *Sox9* and *Sall2* were also detected (Figures S4A and S4B).

**Figure 3:**
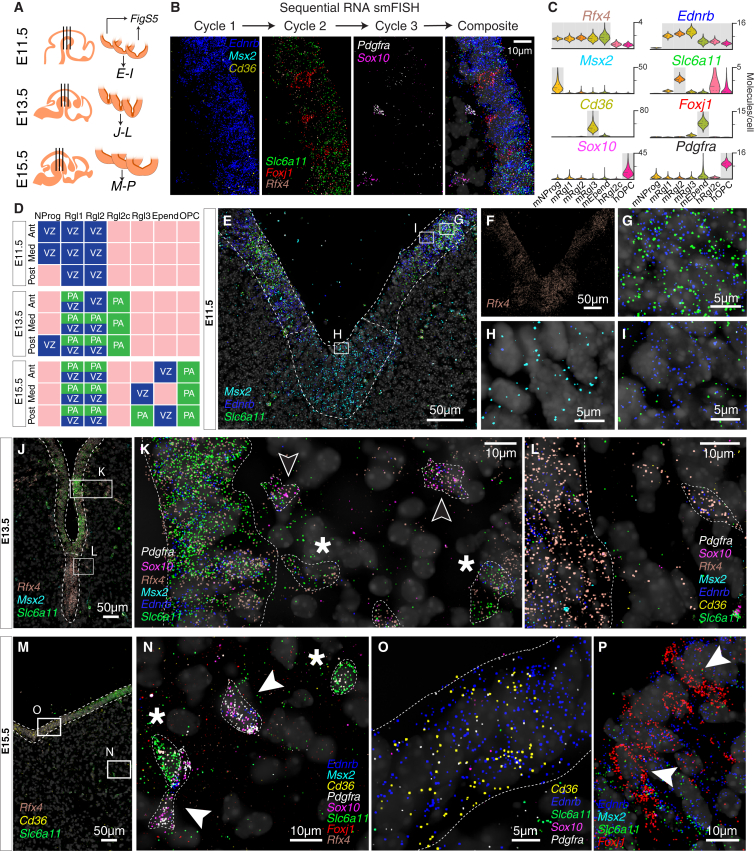
Diversity of Ventricular Zone Cell Types Single-molecule RNA FISH (RNA smFISH) was performed at three anteroposterior positions and three developmental time points, each with eight genes detected in the same single tissue section by sequential hybridizations. For clarity, the panels of this figure show subsets of markers, but all eight markers were stained for in the same single sections. (A) Schematic of coronal sections taken in anteroposterior axis over several developmental time points, not to scale. (B) Representative images of sequential RNA smFISH with eight probes over three cycles and corresponding composite. (C) The expression of genes selected for RNA smFISH in mouse and human VZ cell types shown as violin plots. The violin plots represent the posterior probability distribution for the expressed number of mRNA molecules per cell type. Grey, enriched over baseline with posterior probability >99.8%. Right axis shows absolute molecule counts. (D) Diagram showing the spatiotemporal location of cells discovered by RNA smFISH in the sections shown in (A). VZ, ventricular zone; PA, parenchyma. Representative images are shown in Figure S5. (E) RNA smFISH showing the distribution of *Msx2*, *Ednrb*, and *Slc6a11* in the E11.5 ventral midbrain. (F) Signal for *Rfx4* on the same section as (E), shown separately for clarity. (G) Inset as indicated in (E). (H) Inset as indicated in (E). (I) Inset as indicated in (E). (J) RNA smFISH showing the distribution of *Rfx4*, *Msx2*, and *Slc6a11* in the E13.5 ventral midbrain. (K) Inset as indicated in (J) with additional markers *Pdgfra*, *Sox10*, and *Ednrb*. ^∗^, putative mRgl2 cell; arrow, putative intermediate between Rgl2 and OPC. (L) Inset as indicated in (J) showing markers in (K) in addition of *Cd36*. (M) RNA smFISH showing the distribution of *Rfx4*, *Cd36*, and *Slc6a11* in the E15.5 ventral midbrain. (N) Inset as indicated in (M) with additional markers *Pdgfra*, *Sox10*, *Foxj1*, *Msx2*, and *Ednrb*. ^∗^, putative mRgl2 cell; arrow, putative mOPC located in the parenchyma. (O) Inset as indicated in (M) with additional markers *Pdgfra*, *Sox10*, and *Ednrb* in the VZ. (P) RNA smFISH showing *Foxj1*, *Msx2*, *Slc6a11*, and *Ednrb*. Arrowheads indicate putative ependymal cells in VZ.

**Figure S5:**
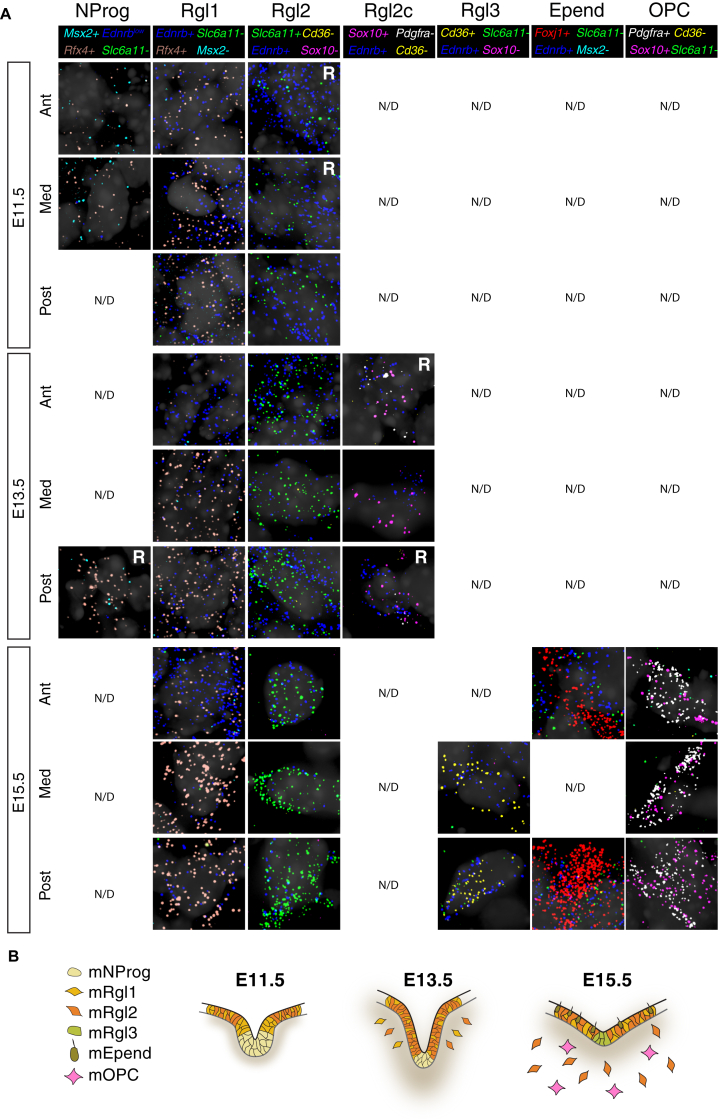
Radial Glia Single Molecule RNA FISH, Related to Figure 3 (A) Representative images corresponding to the diagram in Figure 3D. For clarity, every cell type was identified unambiguously by the expression of two genes and by the non-expression of two other genes. All images correspond to a field of size 13μm × 13μm. (B) Schematic of cell types at different sampled time points.

To examine whether mRgl1–3 cells occupy different compartments of the VZ, we performed multiplexed single-molecule RNA fluorescence in situ hybridization (RNA smFISH) (Lubeck et al., 2014, Lyubimova et al., 2013) using eight genes either shared or specific to different cell types (Figures 3A–3C; Table S3). We stained all eight genes sequentially on the same sections and examined embryos at three developmental stages (E11.5, 13.5, and 15.5) and three anteroposterior levels (Figures 3A, 3D, and S5). The transcription factor *Rfx4,* shared by all five mouse VZ cell types, confirmed the localization of these cells in the VZ (Figure 3F). mNProg cells, identified by *Msx2* expression, were only present in the floor plate, spanning the entire VZ (Figures 3D, 3E, 3H, and S5). In contrast, mRgl2 cells, identified by the expression of *Ednrb* and *Slc6a11*, the GABA reuptake transporter, were confined to the basal plate VZ from E11.5 to E15.5 (Figures 3D, 3E, 3G, 3J, 3K, 3P, and S5). mRgl1 cells (*Rfx4*^*+*^/*Ednrb*^*+*^/*Slc6a11*^*–*^/*Cd36*^*–*^) were observed in both the floor and basal plates (Figures 3D, 3I, 3L, and S5), while *Cd36*^*+*^ mRgl3 were found in the VZ of the floor plate at E15.5, appearing alongside *Foxj1*^*+*^ mEpend cells in this region (Figures 3D, 3M, 3O, 3P, and S5). Combined, these findings demonstrate a previously unsuspected spatial and temporal dynamic arrangement of multiple molecularly diverse radial glia-like cell types.

While mNProg and mEpend cells were confined to the VZ, the somata of mRgl1–3 were found not only in the VZ, but also emerging from the VZ and in the adjacent intermediate and marginal zones, from E13.5 to E15.5 (Figures 3D, 3K, 3L, 3N, and S5), showing that they migrate away from the VZ as it has been described for human cortical outer radial glia (Taverna et al., 2014). Since mRgl2 shared with adult astrocytes the expression of markers such as *Slc6a11*, *TncI*, and *Aldoc* (Cahoy et al., 2008, Hatada et al., 2008, Karus et al., 2011) and radial glia cells can generate astrocytes and oligodendrocytes (Kriegstein and Alvarez-Buylla, 2009, Spassky et al., 2005), we suggest that cells migrating away from the VZ may be initiating gliogenesis.

Notably, our analysis of the human midbrain also identified hOPC, as well as a radial glia-like cell type with expression of some OPC markers (hRgl2c, expressing *OLIG2* and *ETV5;* Figures S2 and S4A). Examination of E13.5 mouse tissue (Figures 3D, 3K, and S5) similarly revealed the presence of cells in the basal plate with radial glia markers, *Ednrb* and *Rfx4*, as well as the OPC marker *Sox10*, but little or no *Pdgfra* (also an OPC marker). Two days later, in the same position, we observed *Pdgfra*^*+*^/*Sox10*^*+*^/*Ednrb*^*+*^ OPCs (Figures 3D, 3N, and S5). These findings suggest that OPCs are likely generated from basal plate radial glia, through a subventricular radial glia-like cell type (Rgl2c) that expresses *Sox10*, but not yet *Pdgfra*, as it emerges from the VZ. Our results thus identify Rgl2c as a subventricular radial glia-like cell type and as a cell type in the oligodendrocyte lineage, linking VZ Rgl with OPCs.

### Neuronal Progenitors, Neuroblasts, and Non-Dopaminergic Neurons

The diversity of neurons in the ventral midbrain is generated as a result of patterning events that define spatial domains of differentiation. While only one neuronal progenitor (mNProg) was found in the mouse VZ, five different progenitors were identified in human (hNProg; hProgM [midline progenitor]; hProgFPM [medial floorplate progenitor]; hProgFPL [lateral floorplate progenitor]; hProgBP [basal plate progenitor]). All human progenitors expressed *HMGA1* and *HMGB2* and shared with hRgl1 the expression of *OTX2* (which distinguishes the forebrain and midbrain from the hindbrain [Acampora et al., 1995]). The mouse midline marker *CORIN1* (Ono et al., 2007) was expressed together with *TOX*, a transcriptional regulator of Sox2 (Artegiani et al., 2015), in hProgM. Floorplate progenitors were identified by the expression of *LMX1A*, a transcription factor that specifies dopaminergic neurons (Andersson et al., 2006) and labels the human floorplate (Hebsgaard et al., 2009). High levels of the morphogen *WNT1*, expressed in the lateral aspect of the floorplate at E10.5 (Prakash et al., 2006), identified hProgFPL and hProgFPM (low *WNT1* levels; Figure S2). Laterally, hProgBP were found to express *FOXA2* and *DMBX1*, but not *WNT1* or *LMX1A* (Figures S2 and S4A). These four progenitors also expressed *CNPY1*, a positive regulator of FGF signaling in the midbrain-hindbrain region (Hirate and Okamoto, 2006). Lastly, hNProg expressed pro-neurogenic genes such as *NEUROG1* and shared with the hNbM the expression of *NEUROD1*, *NEUROD4*, *NEUROG2*, and *NHLH1* (Figure S4A), indicating an active role in neurogenesis. These findings show that molecularly defined human progenitor cell types correspond to previously defined ventral midbrain domains in mouse.

**Figure 4:**
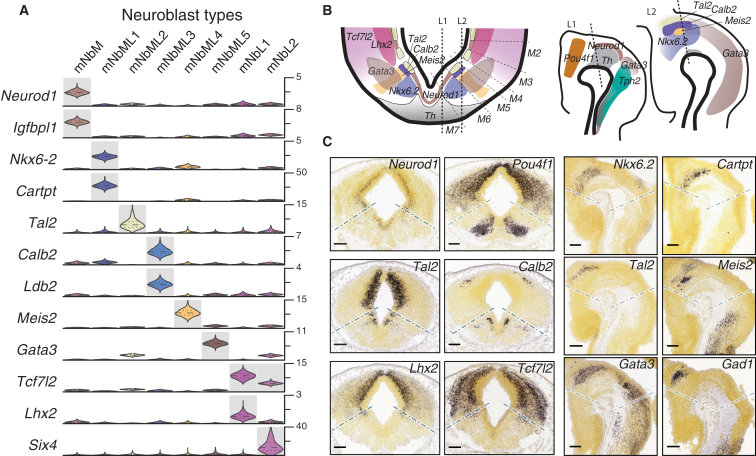
Neuroblast Patterning (A) Mouse neuroblast populations and their markers. Grey, enriched over baseline with posterior probability >99.8%. (B) Simplified illustration of the patterning of the ventral midbrain, based on transcriptionally defined domains. Left, coronal view; right, sagittal views at the indicated levels (L1 and L2). (C) In situ hybridization (image data from Allen Institute for Brain Science: Allen Developing Mouse Brain Atlas) for key domain-specific transcription factors and markers. Dashed lines indicate the approximate extent of the ventral region used in our dissections (scale bar, 200 μm).

Among mouse neuroblasts, we identify eight cell types that were very similar but with each expressing known transcription factors that defined their spatial position in the anterior/posterior and medial/lateral axes as well as markers that are maintained in the neuronal types they give rise to (Figures 4A–4C). For instance, mNbM expressed a nuclear receptor required for dopaminergic neuron development, *Nr4a2* (known as *Nurr1*; Zetterström et al., 1997). Mediolateral neuroblasts type 1 (mNbML1) expressed *Cartpt,* as well as *Nkx6-2*, which is found in the intermediate zone domain where OMTN and RN neurons appear (Prakash et al., 2009), in the midbrain domain m6 (Nakatani et al., 2007). We also found mediolateral neuroblasts capable of giving rise to GABAergic neurons (Achim et al., 2013), such as *Tal2*^*+*^ mNbML2 in m5 and m3-1, and *Gata3*^*+*^ mNbML5 in m5-m3.

**Figure S6:**
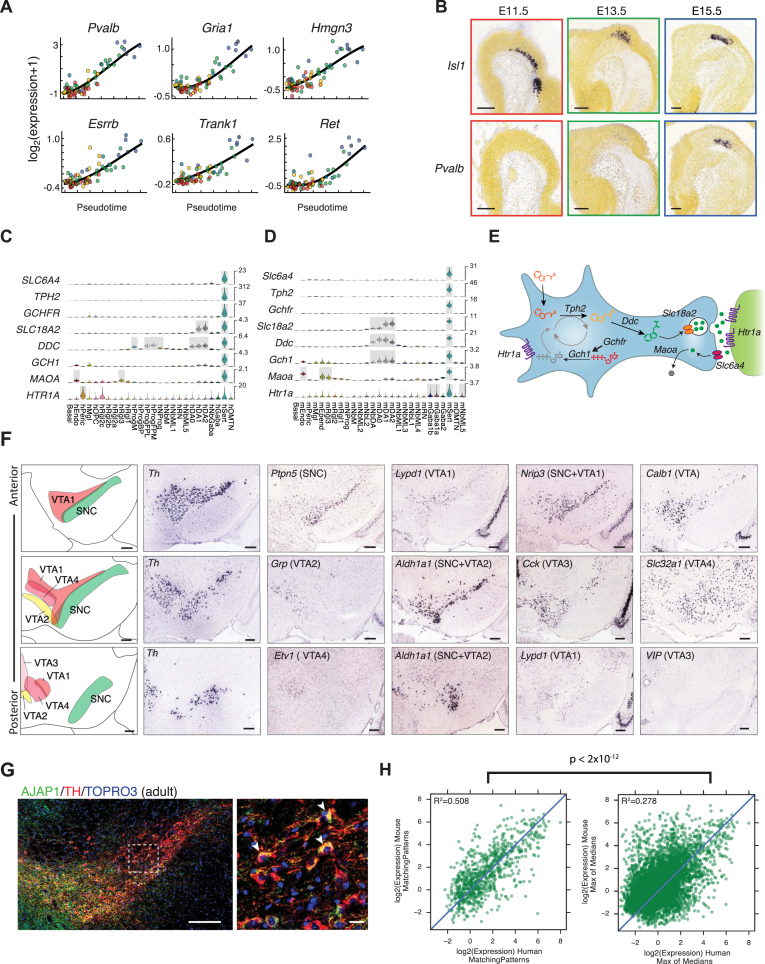
Oculomotor and Trochlear, Serotonergic, and Dopaminergic neurons, Related to Figure 4 and 6 (A) Examples of genes regulated along pseudotime in mOMTN. No genes were significantly downregulated. (B) Validation of the induction of *Pvalb* during maturation of mOMTN, shown alongside *Isl1* in sagittal sections of mouse embryos (in situ hybridization data from the Allen Developing Mouse Brain Atlas) (scale bars 200 μm). (C) Violin plots showing the expression of key genes involved in serotonergic synapse function, across all human cell types. (D) Violin plots showing the expression of key genes involved in serotonergic synapse function, across all mouse cell types. (E) Schematic of the function of genes in (C/D) in a serotonergic neuron, here drawn in place of their corresponding protein products (adapted from Deneris and Wyler, 2012). (F) Spatial distribution of the five adult dopaminergic cell types (in situ hybridization data from Allen Mouse Brain Atlas) (scale bars 200 μm). (G) Validation of AJAP1 as a pan-dopaminergic marker in the adult mouse brain (scale bar left 100 μm; scale bar right 20 μm). (H) Scatterplots showing the level of expression of genes expressed above baseline in matching cell types (left) and the correlation of the cell types that express the genes at higher levels (right)

The progressive changes in gene expression profiles from neuroblasts to mature neurons led us to think that neuronal differentiation may mainly involve gain of gene expression. We tested this idea in mOMTNs, which were present already at early time points (identified by fundamental transcription factors such as *Isl1*, *Lhx4*, *Phox2a*, *Phox2b*, and *Tbx20*; Figure S3). A pseudotime analysis over developmental time (Figure S6A) showed that late motorneurons acquired the expression of *Pvalb* (Figure S6B), as well as genes such as *Gria1*, *Hmgn3*, *Esrrb*, *Trank1*, and *Ret*. In fact, 121 genes were significantly upregulated, but not a single gene was downregulated (STAR Methods). Similarly, mSert and hSert were characterized by highly specific induction of the key genes necessary for serotonergic synaptic function (Deneris and Wyler, 2012) (Figures S6C–S6E). Thus, after the acquisition of a basic neuronal identity, maturation appears to mainly involve the addition of genes with cell-type-specific functions.

### Diversity in Mouse and Human Embryonic Dopaminergic Neuron Development

**Figure 5:**
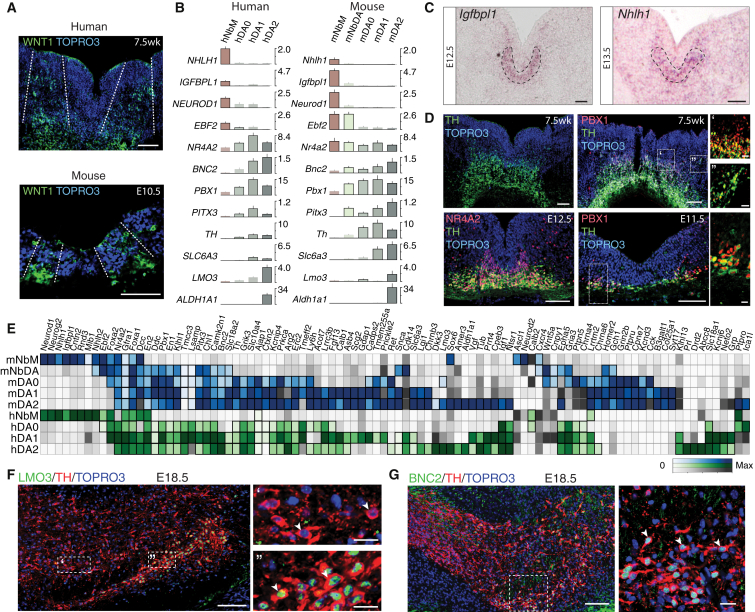
Comparison of Mouse and Human Dopaminergic Neuronal Development (A) WNT1 compartments marking lateral population of the floor plate in human and mouse tissue (scale bar, 100 μm). (B) Bar plot of cell types of the human and mouse dopaminergic lineage, showing the expression of key genes. Bars show average mRNA expression, scaled to the absolute molecule counts indicated on the right axis. Error bars show SEM. (C) Validation of mNbM by in situ hybridization for *Igfbpl1* and *Nhlh1* (scale bar, 50 μm). (D) Neuroblasts in human and mouse ventral midbrain (scale bar, 100 μm; magnification, 20 μm). (E) Selected genes showing similar (left) or distinct (right) expression in mouse and human ventral midbrain. Blue, expressed above baseline in mouse (>99.8% posterior probability); green, expressed above baseline in human (>99.8% posterior probability); gray, not expressed above baseline. (F) Validation of LMO3 expression by immunohistochemistry in a subset of TH+ neurons in the E18.5 mouse ventral midbrain (scale bar left, 100 μm; right, 20 μm). (G) Validation of BNC2 expression by immunohistochemistry in TH^+^ neurons in the E18.5 mouse ventral midbrain (scale bar left, 100 μm; right, 20 μm).

Focusing on the dopaminergic lineage revealed both similarities and differences between species. Similarities included that floorplate progenitors found in human were also present in mouse, at E10.5, as assessed by the presence of Wnt1 in lateral floorplate progenitors (Figure 5A; Prakash et al., 2006). The first postmitotic cell in the dopaminergic lineage in both mouse and human, NbM, expressed not only *Nr4a2*, but also *Igfbpl1* and transcription factors such as *Neurod1*, *Neurod2*, *Klf12*, and *Nhlh1* (Figures 5B and 5C). Notably, NbM and NProg in both species also shared the expression of *Neurog2*, a proneural gene required for dopaminergic neurogenesis (Kele et al., 2006).

We also found three distinct types of embryonic dopaminergic neurons in both mouse and human (Figure 5B): (1) a very immature DA0, which expressed tyrosine hydroxylase (*Th*), the rate-limiting enzyme in the synthesis of dopamine, in addition to the factors above; (2) DA1 neurons, which additionally expressed the dopamine transporter, *Slc6a3*; and (3) DA2 neurons, distinguished by the specific addition of *Aldh1a1* and the transcriptional co-regulator *Lmo3*, a LIM domain only protein that interacts with basic-loop-helix proteins to regulate neurogenesis and has been involved in the specification of hippocampal neurons (Bao et al., 2000, Hinks et al., 1997). In addition, we identified and validated two genes conserved in mouse and human dopaminergic neurons: *Bnc2*, expressed in all dopaminergic neurons, and *Lmo3*, expressed in subset a of TH^+^ cells (Figures 5E–5G).

However, a number of differences in mouse and human dopaminergic neuron development were also identified. First, key transcription factors such as *Msx1 and Lmx1a*, the latter required for the specification of dopaminergic neurons (Andersson et al., 2006), did not appear in the same cell types: mNProg (Figure S3) versus hRgl1 (Figure S2). Second, an intermediate neuroblast, mNbDA (expressing *Pbx1*, but not *Th*) was found in mouse, but not in human (Figure 5D). Third, *Ebf2*, a transcription factor regulating different aspects of dopaminergic neuron development (Yang et al., 2015, Yin et al., 2009), was found in the two mouse neuroblasts (mNbM and mNbDA), but in human, it appeared later, in immature dopaminergic neurons (hDA0). The same was true for transcription factors expressed in mNbDA, such as *Pbx1* and *Pitx3*, both required for dopaminergic development (Nunes et al., 2003, Villaescusa et al., 2016), which were only found in hDA0 (Figures 5B and 5D). Fourth, a detailed analysis of gene expression in human and mouse dopaminergic neuroblasts and neurons (Figure 5E) revealed notable species differences in gene expression. For instance, *Cck*, *Grin2b*, and *Homer2* were not detected in human cells, and specific ion channels present in human, such as *KCNJ6*, were not detected in rodent (Figure 5E).

### Adult Ventral Midbrain Dopaminergic Neuron Subtypes Emerge Postnatally

**Figure 6:**
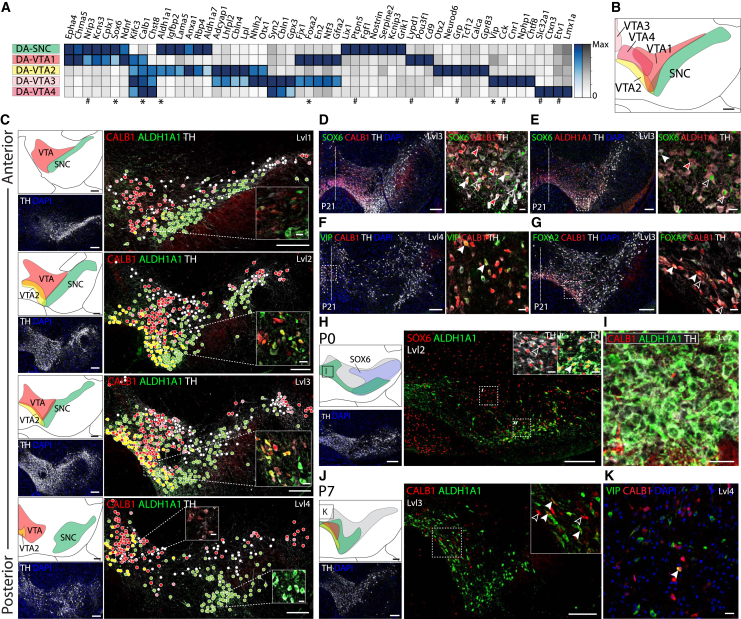
Diversity of Adult Dopaminergic Neurons (A) Expression of selected genes across five adult dopaminergic cell types. Colors as in (5E). ^∗^, indicates selected genes for validation in (C–K). #, indicates genes selected for in situ validation from Allen Mouse Brain Atlas found in Figure S6F. (B) Projected schematic of five distinct dopaminergic populations in adult ventral midbrain. (C) Anterior to posterior representation of dopaminergic neuron populations at P21. Detection of CALB1, ALDH1A1, and TH. Green dots, TH^+^/ALDH1A1^+^/CALB1^−^; Yellow dots, TH^+^/ALDH1A1^+^/CALB1^+^; Red/Pink (high/low expression) dots, CALB1^+^/TH^+^/ALDH1A1^−^; White dots, TH^+^. (scale bar, 200 μm; insets, 20 μm). (D) Validation of dopaminergic neurons at P21. Open arrow, SOX6^+^/CALB1^−^ (SNC); solid white arrow, SOX6^+^/CALB1^+^ (VTA1); solid red arrow, SOX6^−^/CALB1^+^ (VTA2-4) (scale bar left, 200 μm; right, 20 μm). (E) Validation of dopaminergic neurons at P21. Open arrow, SOX6^+^/ALDH1A1^+^ (SNC); solid white arrow, SOX6^−^/ALDH1A1^−^ (VTA3/4); solid red arrow, SOX6^−^/ALDH1A1^+^ (VTA2) (scale bar left, 200 μm; right, 20 μm). (F) Validation of dopaminergic neurons at P21 in posterior section. Solid white arrow VIP+/CALB1+ (VTA3) (scale bar left, 200 μm; right, 20 μm). (G) Validation of dopaminergic neurons at P21. Open arrow, FOXA2^−^/CALB1^+^ (VTA2/4); solid white arrow, FOXA2^+^/CALB1^+^ (VTA1/3) (scale bar left, 200 μm; right, 20 μm). (H) SOX6^+^/ALDH1A1^+^ visible at P0. Insets: open arrow, SOX6^+^/ALDH1A1^−^ (putative VTA1); solid white arrow, SOX6^+^/ALDH1A1^+^ (SNC) (scale bar, 20 μm). (I) Absence of CALB1^+^ in TH^+^/ALDH1A1 cells at P0 (scale bar, 20 μm). (J) CALB1^+^ (open arrow) and CALB1^+^/ALDH1A1^+^ (solid arrow, VTA2) visible at P7 (scale bar, 200 μm; insets, 20 μm). (K) VIP^+^/CALB1^+^ (solid arrow, VTA3) visible at midline at the posterior level of P7 (scale bar, 20 μm).

Previous work by single-cell qPCR on FACS-sorted SLC6A3^+^ cells (Poulin et al., 2014) identified five types of dopaminergic neurons postnatally. We confirmed this finding in a separate single-cell RNA-seq analysis of adult mouse dopaminergic neurons (Figures 6A and 6B). The presence of the pan-dopaminergic marker, AJAP1, in TH^+^ cells was validated by immunohistochemistry (Figure S6G). One cell type mapped to the SNc (mDA-SNC) and the rest mainly to distinct regions of the VTA (mDA-VTA1, 2, and 4) and the periaqueductal gray (mDA-VTA3) (Figures 6C–6G and S6F). We validated these adult dopaminergic neuron populations and determined their positions using the Allen Mouse Brain Atlas (Figure S6F) and by immunohistochemistry with multiple antibodies (Figures 6C–6G).

The presence of five dopaminergic cell types in the adult, and only two in the embryo, led us to examine the postnatal maturation of this lineage. We noted that some genes expressed in embryonic mDA2 neurons at E18.5, such as *Aldh1a1*, *Sox6*, and *Calb1*^*Low*^, were later found in mDA-SNC, mDA-VTA1, and mDA-VTA2 neurons, suggesting that mDA2 neurons could be a common ancestor. Immunohistochemical analysis of ALDH1A1 and SOX6 during postnatal development revealed the presence of distinct subpopulations already at P0 (Figure 6H), suggesting that mDA2 neurons were already then maturing into DA-SNC. However, the shared VTA marker CALB1 was only detected at P7 (Figures 6I and 6J), indicating that their maturation into proper DA-VTA cells occurs subsequently. At this stage, TH, CALB1, ALDH1A1, and SOX6 allowed us to distinguish VTA1, VTA2, and SNc cells from other VTA cells (Figure 6J). The first VIP^+^/CALB1^+^ cells were also detected at P7 (Figure 6K), indicating that further VTA subdivisions, such as DA-VTA3, also emerge at this point. Combined, our findings show that adult dopaminergic subtypes emerge postnatally as a result of environmental cues rather than early patterning events.

### Stem-Cell-Derived In Vitro Dopaminergic Neurons

Cell replacement therapy is one of the most promising future treatments for Parkinson’s disease. Transplantation of human fetal midbrain tissue containing dopaminergic neurons has provided proof of principle for this therapeutic approach (Barker et al., 2015). More recently, by providing some of the key developmental signals that control midbrain dopaminergic neuron development (Arenas et al., 2015), it has become possible to generate human pluripotent stem cell (hPSC)-derived dopaminergic neurons, capable of inducing behavioral recovery in animal models of Parkinson’s disease (Kirkeby et al., 2012, Kriks et al., 2011). However, the molecular composition of hPSC-derived preparations at a single-cell level is completely unknown, and as the field is moving closer to clinical trials (Barker et al., 2015), it will be important to determine whether the cell types present in hPSC-derived cell preparations actually resemble their in vivo counterparts.

We used our comprehensive human reference dataset to assess the composition of such stem cell preparations at different stages of differentiation, as well as the fidelity of in vitro derived dopaminergic neurons as compared with those found in vivo.

**Figure 7:**
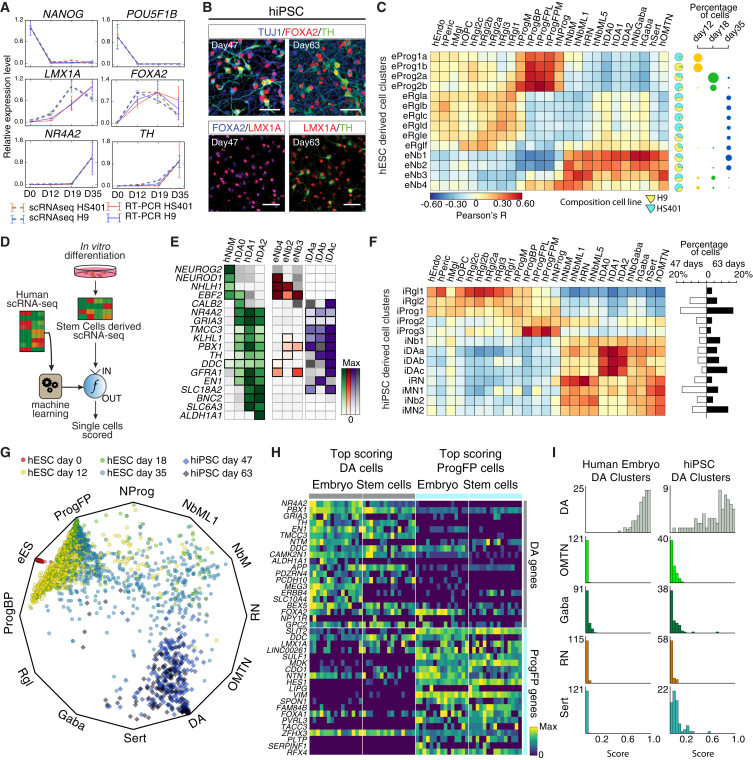
Single-Cell Analysis of Differentiated In Vitro Human ESC and iPSC Cultures and Prototype-Based Scoring (A) Expression of marker genes during the differentiation protocol measured by qPCR (solid lines) or inferred by pooling of single-cell RNA-seq data per time point (dashed lines). Red, HS401 cells; blue, H9 cells. (B) Immunostaining of hiPSC cultures (scale bar, 50 μm). (C) hESC-derived cell types compared with in vivo cell types. Heatmap shows correlation based on genes that show specific expression in any in vivo cell type. Pie chart (right) shows relative contribution of each cell line to each cluster. Dot plot shows distribution of cells at different time points. (D) Schematic of the inference of cell identity using prototype scoring. A machine learning model was trained on in vivo cell types (left) resulting in a reusable scoring function. Stem cells were then scored and evaluated individually, without the need for clustering. Typical visualization outputs of the machine learning model are shown in (G–I). (E) Expression of selected genes of the dopaminergic lineage in human development, hESC-, and hiPSC-derived cell types. (F) hiPSC-derived cell types compared with in vivo cell types. Heatmap shows correlation based on all genes that show specific expression in any in vivo cell type. Bars (right) show relative abundances at 47 and 63 days in vitro. (G) Wheel plot showing the prototype scores for hESCs and hiPSCs. Dots represent individual cells and the distance to each prototype is proportional to the relative score of that prototype. Colors indicate time point of differentiation (red, day 0; dark blue, day 63). (H) Heatmap showing gene expression of top-scoring dopaminergic (left) and floorplate progenitor (right) cells. Each subpanel shows individual in vivo (left in each subpanel) and in vitro (right in each subpanel) cells. Genes shown have the top 20 highest weights for the scoring of dopaminergic or floorplate progenitor, respectively. (I) Histograms of prototype scores for human dopaminergic clusters and hiPSC-derived dopaminergic clusters.

**Figure S7:**
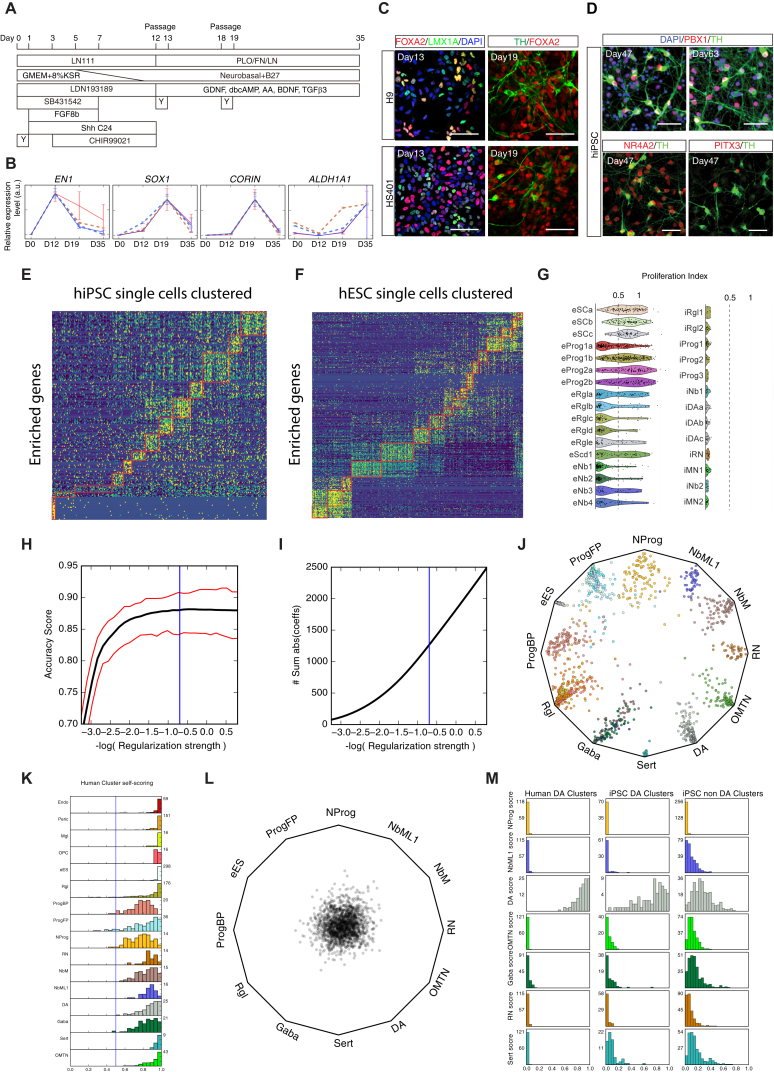
Stem Cells Differentiation Protocol and Machine Learning Performance and Diagnostics, Related to Figure 7 (A) Schematic of the hESCs in vitro differentiation protocol. (B) Variation of expression of marker genes during the differentiation protocol measured by qPCR. As a comparison, values calculated by summing single cell expression levels are shown as dashed line. (C) Immunostaining of hESC cultures (scale bar 50 μm). (D) Immunostaining of hiPSC cultures (scale bar 50 μm). (E) Heatmap showing raw hiPSCs data clustered. Columns are single cell and rows genes. Cell clusters and genes enriched in every cluster are surrounded by red boxes. (F) Heatmap showing raw hESCs data as in (E). (G) Violin plots showing proliferation index distribution for each hiPSC and hESC cluster. (H) Line plot showing the accuracy score of the classifier varying with decreasing regularization strength as estimated by cross-validation. Red line shows 95% C.I. on the estimation of the accuracy score. (I) Line plot showing the total absolute values of weights. Blue vertical line in (H-I) shows the chosen regularization parameter. (J) Training dataset plotted on wheel plot as in Figure 7G. (K) Score distribution on the training dataset clusters. (L) Negative control cells are obtained by scrambling gene values of cells of the training dataset and are plotted on wheel plot as in Figure 7G. (M) Histograms showing classifier scores of single cells belonging to the human dopaminergic clusters (left), to the dopaminergic hiPSC clusters (center) and to the rest of the cells in the hiPSC preparation (right).

Human embryonic stem cells (hESCs) and human induced pluripotent stem cells (hiPSCs) were differentiated using the protocol by Kriks et al. (2011) (Figure S7A). We first analyzed the hESC (H9 and HS401 lines) cultures using classical methods such as qPCR (Table S4) and immunohistochemistry. ESC markers *NANOG* and *POU5F1* expression disappeared by day 12. Examining genes required for dopaminergic neuron differentiation, we found that *FOXA2* peaked at day 19, *LMX1A* progressively increased from day 12 to 35, and *NR4A2* as well as *TH* increased between day 19 and 35 (Figures 7A, S7B, and S7C). Immunohistochemistry revealed that by day 47 most hiPSC-derived cells were positive for neurofilament (TUJ1; a marker of mature neurons), and their processes were much longer at day 63. At day 47, most cells were positive for both FOXA2 and LMX1A, with 85% of the cells in the culture being FOXA2^+^ and 21% TH^+^ (Figure 7B). In addition, TH^+^ cells were NR4A2^+^, PBX1^+^, and some expressed PITX3^+^ (Figure S7D), indicating that they differentiated along the dopaminergic lineage.

Single-cell RNA-seq was performed on cells obtained from hESCs (H9 and HS401 lines) at days 0, 12, 18, and 35, as well as hiPSCs at days 47 and 63. Pooled single-cell data closely recapitulated the bulk expression levels of the genes previously analyzed by qPCR (Figures 7A and S7B), confirming that no major cell type was lost in the single-cell analysis of these cultures.

Clustering revealed the presence of 14 hESC-derived (Figures 7C and S7F) and 13 distinct hiPSC-derived cell types (Figures 7F and S7E), which resembled some of the 25 human neural cell types present in the human fetal midbrain tissue. hESC-derived cultures generated a range of poorly defined radial glia-like cells and progenitors that resembled those in the floorplate and basal plate (Figures 7C and S7G). hESC cultures also generated four types of neuroblasts from day 12 to 35, resembling hNbM, hNbML1, and hNbGaba. hiPSCs, differentiated for 47 and 63 days, gave rise to more mature cells, including two radial glia-like cell types and three progenitors (Figure 7F). hiPSCs also generated two types of neuroblasts, two motor-neuron-like cell types as well as one well-defined red-nucleus-like neuron and three hiPSC-derived dopaminergic cell types (iDAa-c) with features of human fetal dopaminergic neurons (hDA0-2). The most mature dopaminergic cell type (iDAc) expressed key genes such as *NR4A2*, *KLHL1*, *PBX1*, *SLC18A2*, *TH*, *DDC*, *GFRA1*, or *EN1* (Figure 7E). We conclude that these preparations contain a cell type diversity much greater than previously known, including *TH*^+^ cells at a stage of differentiation similar to the tissue currently used for cell transplantation in Parkinson’s disease.

To assess the quality of the in vitro differentiated cells, we developed a machine-learning tool that compares the transcriptomes of each in vitro cell to the cell types found in vivo (Figures 7D, S7H, S7I, and S7L; STAR Methods). This approach allows visualizing each individual cell based on the probability of being each of the prototypical cell types (Figures 7G and S7J). While endogenous midbrain cells showed distinct, unambiguous identities (Figures S7J and S7K), hPSC-derived cells showed more intermediate forms (Figure 7G). However, a clear trajectory was observed over time from pluripotent state (day 0), through progenitors (day 12 and 18), medial and mediolateral neuroblasts (day 18 and 35), and finally converging on dopaminergic cells at days 47 and 63. Thus, stem cells under these culture conditions recapitulated key stages of in vivo ventral midbrain development.

Comparing the fifteen highest-quality progenitor-like (ProgFP) and dopaminergic-like neurons to their in vivo counterparts (Figure 7H) revealed accurate expression of key developmentally regulated genes. For example, both embryonic and in vitro dopaminergic neurons expressed *NR4A2*, *PBX1*, *EN1*, *TH*, and *DDC*, which were not found in progenitors. Similarly, both in vitro and in vivo progenitors expressed *VIM*, *HES1*, *SLIT2*, and *RFX4*, which were not found in mature neurons. However, the in vitro cells differed from the in vivo prototypes in global expression profiles and scores (Figures 7I and S7M) indicating that further improvement of differentiation protocols is possible.

In sum, our analysis identifies not only genes and cell types in mouse and human ventral midbrain development, but also provides tools to evaluate gene expression in stem-cell-derived dopaminergic preparations, determine their quality, and guide future improvements in Parkinson’s disease cell replacement therapy. We propose this approach as a preferred strategy for assessing the quality of stem cell preparations for clinical applications.

## STAR★Methods

### Key Resources Table

**Table:** 

REAGENT or RESOURCE	SOURCE	IDENTIFIER
**Antibodies**		

Rabbit polyclonal anti-FOXA2	Cell Signaling Technology	Cat#3143S; RRID: AB_2104878
Rabbit polyclonal anti-LMX1	Merck Millipore	Cat#AB10533; RRID: AB_10805970
Sheep polyclonal anti-TH	Novus Biologicals	Cat#NB300-110; RRID: AB_10002491
Rabbit polyclonal anti-TH	PelFreez Biologicals	Cat#P40101; RRID: AB_2313713
Mouse monoclonal anti-βIII Tubulin	Promega	Cat#G7121; RRID: AB_430874
Goat polyclonal anti-NURR1	R&D Systems	Cat#AF2156; RRID: AB_2153894
Goat polyclonal anti-LMO3 (C-14)	Santa Cruz Biotechnology	Cat#sc-82647; RRID: AB_2136576
Mouse monoclonal anti-PBX1a (710.2)	Santa Cruz Biotechnology	Cat#sc-101851; RRID: AB_2299285
Goat polyclonal anti-PITX3 (N-20)	Santa Cruz Biotechnology	Cat#sc-19307; RRID: AB_2165313
Rabbit polyclonal anti-BNC2	Sigma-Aldrich	Cat#HPA018525; RRID: AB_1233560
Rabbit polyclonal anti-AJAP1	Sigma-Aldrich	Cat#HPA012157; RRID: AB_2289413
Mouse monoclonal anti-TH	Sigma-Aldrich	Cat#T2928; RRID: AB_477569
Mouse monoclonal anti-Calbindin-D-28K	Sigma-Aldrich	Cat#C9848 RRID: AB_476894
Rabbit polyclonal anti-ALDH1A1	Abcam	Cat#ab23375 RRID: AB_2224009
Rabbit polyclonal anti-WNT1	Abcam	Cat#ab15251 RRID: AB_301792
DAPI	Sigma-Aldrich	Cat#D9542
TO-PRO-3 Iodide	Thermo Fisher Scientific	Cat#T3605
Donkey anti-Rabbit Alexa Fluor 350 secondary	Thermo Fisher Scientific	Cat#A10039 RRID: AB_11180201
Donkey anti-Rabbit Alexa Fluor 488 secondary	Thermo Fisher Scientific	Cat#A21206 RRID: AB_141708
Donkey anti-Rabbit Alexa Fluor 555 secondary	Thermo Fisher Scientific	Cat#A31572 RRID: AB_10562716
Donkey anti-mouse Alexa Fluor 488 secondary	Thermo Fisher Scientific	Cat#A21202 RRID: AB_141607
Donkey anti-mouse Alexa Fluor 555 secondary	Thermo Fisher Scientific	Cat#A31570 RRID: AB_2313501
Donkey anti-mouse Alexa Fluor 647 secondary	Thermo Fisher Scientific	Cat#A31571 RRID: AB_2313501
Chicken anti-goat Alexa Fluor 488 secondary	Thermo Fisher Scientific	Cat#A21467 RRID: AB_10055703
Donkey anti-goat Alexa Fluor 555 secondary	Thermo Fisher Scientific	Cat#A21432 RRID: AB_10053826
Donkey anti-sheep Alexa Fluor 488 secondary	Thermo Fisher Scientific	Cat#A11015 RRID: AB_10561557
Donkey anti-sheep Alexa Fluor 647 secondary	Thermo Fisher Scientific	Cat#A21448 RRID: AB_10374882
Goat anti-guinea pig Alexa Fluor 488 secondary	Thermo Fisher Scientific	Cat#A11073 RRID: AB_2307359
Rabbit polyclonal anti-VIP	Abcam Gift: Ulrika Marklund	Cat#ab43841; RRID: AB_778831
Guinea pig anti-SOX6	Gift: Jens Hjerling-Leffler	N/A

**Chemicals, Peptides, and Recombinant Proteins**		

Human recombinant laminin-521	BioLamina	Cat#LN521
Human recombinant laminin-111	BioLamina	Cat#LN111
Laminin mouse protein	Thermo Fisher Scientific	Cat#23017015
Laminin	Sigma-Aldrich	Cat#L2020
NutriStem hPSC XF Medium	Biological Industries	Cat#05-100-1A
Hibernate-E Medium	Thermo Fisher Scientific	Cat#A1247601
N-2 Supplement	Thermo Fisher Scientific	Cat#17502048
B-27 Supplement	Thermo Fisher Scientific	Cat#17504044
Neurobasal Medium	Thermo Fisher Scientific	Cat#21103049
Glasgow’s MEM (GMEM)	Thermo Fisher Scientific	Cat#11710035
KnockOut Serum Replacement	Thermo Fisher Scientific	Cat#10828028
MEM non-essential amino acids	Thermo Fisher Scientific	Cat#11140-050
Fibronectin	Sigma-Aldrich	Cat#F0895
TrypLE Select Enzyme	Thermo Fisher Scientific	Cat#12563011
Poly-L-ornithine solution	Sigma-Aldrich	Cat#P4957
CHIR99021	Sigma-Aldrich	Cat#SML1046
Ascorbic acid	Sigma-Aldrich	Cat#A4544
dbcAMP	Sigma-Aldrich	Cat#D0627
Y-27632	Tocris	Cat#1254
SB 431542	Tocris	Cat#1614
LDN193189	Stemgent	Cat#04-0074
Recombinant human SHH (C24II)	R&D Systems	Cat#1845-SH-100/CF
Recombinant human FGF8b	R&D Systems	Cat#423-F8/CF
Recombinant human BDNF	R&D Systems	Cat#248-BD-025/CF
Recombinant human GDNF	R&D Systems	Cat#212-GD-050/CF
Recombinant human TGFβ3	R&D Systems	Cat#243-B3
SP6 RNA polymerase	Thermo Fisher Scientific	Cat#EP0133
DIG RNA Labeling Mix	Roche	Cat#11277073910
Anti-Digoxigenin-AP, Fab fragments	Roche	Cat#11093274910 RRID: AB_514497
NBT/BCIP Stock Solution	Roche	Cat#11681451001

**Critical Commercial Assays**		

Papain Dissociation System	Worthington Biochemical	Cat#LK003150
RNeasy Mini Kit	QIAGEN	Cat#74104

**Deposited Data**		

Single-cell RNA-sequencing raw data files	NCBI GEO	GEO: GSE76381

**Experimental Models: Cell Lines**		

Human: H9	Thomson et al., 1998	N/A
Human: HS401	Rodin et al., 2014	N/A
iCell DopaNeurons Kit	Cellular Dynamics International	Cat#DNC-301-030-001

**Experimental Models: Organisms/Strains**		

Mouse: Crl:CD1(ICR)	Charles River	N/A
Mouse: B6.SJL-*Slc6a3*^*tm1.1(cre)Bkmn*^/J	Jackson Laboratories	RRID: IMSR_JAX:006660
Mouse: B6.*Cg-Gt(ROSA)*^*26Sortm14(CAG-tdTomato)Hze*^/J	Jackson Laboratories	RRID: IMSR_JAX:007914

**Sequence-Based Reagents**		

C1-P1-PCR2	Islam et al., 2014	N/A
C1-TN5-U	Islam et al., 2014	N/A
C1-P1-T31	Islam et al., 2014	N/A
C1-P1-RNA-TSO	Islam et al., 2014	N/A
Primers for single-molecule RNA FISH; Table S3	This paper	N/A
Primers for qPCR; Table S4	This paper	N/A
In Situ Forward Primer, *Nhlh1*, TGT TCA GCC ACA AGC TGC	This paper	N/A
In Situ Reverse Primer, *Nhlh1*, GAG ATT TAG GTG ACA CTA TAG AGC GCT CCT CAC GAC TCA A	This paper	N/A
In Situ Forward Primer, *Igfbpl1*, TCA CCT TGC ATG AAC AGC TCA G	This paper	N/A
In Situ Reverse Primer, *Igfbpl1*, GAG ATT TAG GTG ACA CTA TAG ACT TGC CCA GGG TCA TAC AG	This paper	N/A

**Software and Algorithms**		

Cell-scoring command-line tool	This paper	http://github.com/linnarsson-lab/ipynb-lamanno2016/tree/master/scoringtool
Ipython notebooks showing key steps of the analysis	This paper	http://github.com/linnarsson-lab/ipynb-lamanno2016
BackSPIN algorithm	Zeisel et al., 2015	https://github.com/linnarsson-lab/BackSPIN

### Contact for Reagent and Resource Sharing

Further information and requests for reagents may be directed to, and will be fulfilled by the Lead Contact Sten Linnarsson (sten.linnarsson@ki.se).

### Experimental Model and Subject Details

#### Mice

Wild-type CD-1 mice were obtained from Charles River (Germany). CD-1 mice were mated overnight and noon of the day was considered E0.5 and then shipped as pregnant females. Mice were housed in rooms with regular dark/light cycle and fed standard rodent diet and water ad libitum. Mice were housed in groups up to four animals on saw dust bedding and straw for nest building. For postnatal animals, the day mice were born was considered P0. A DAT1-Cre driver line (Bäckman et al., 2006) was crossed with a floxed tdTomato reporter strain (Madisen et al., 2010), resulting in a *DAT1-Cre/tdTomato* mouse strain expressing tdTomato in DAT1 (dopamine transporter, *Slc6a3*) positive neurons. Mice were housed and tissue obtained following guidelines and permissions from the local ethics committee, Stockholm Norra Djurförsöksetisks Nämd (N326/12), and Swiss National and Institutional guidelines.

#### Human tissue

Human fetal tissues were collected from routine termination of pregnancies at Addenbrooke’s Hospital (Cambridge) and dissected in HIBERNATE media. Samples for single cell analysis were screened for biohazards and then shipped overnight on ice (in HIBERNATE media) to Sweden. Ethical approval for the use of postmortem human fetal tissue was provided by the National Research Ethics Service Committee East of England - Cambridge Central (Local Research Ethics Committee, reference no. 96/085).

#### Cell lines

hiPSC-derived dopaminergic neurons (iCell DopaNeurons) were obtained from Cellular Dynamics International (lot#6003358) were cultured following the manufacturer’s recommendations. Briefly, cells were thawed and plated on PLO and Laminin coated plates with media and supplements provided by manufacturer. Cells were cultured in a 37°C, 5% CO_2_ incubator, and media was exchanged every 2-3 days.

Human ESCs H9 **(**passage 46-49) (Thomson et al., 1998) and HS401 (passage 40-43) (Rodin et al., 2014) were maintained on a recombinant human laminin-521 (BioLamina) coated dish with NutriStem hESC XF (Biological Industries). These cells were passaged at 1:10-1:20 ratio for each passaging (Rodin et al., 2014). The cell lines were authenticated as ES cells by qPCR and immunohistochemistry using a standard panel of stem cell markers and dopaminergic lineage markers.

### Method Details

#### Tissue collection

CD-1 mice were sacrificed and embryos were dissected out of the uterine horn between the time points E11.5 and E18.5. Between 6-16 ventral midbrain tissue pieces (see Table S1) were dissected from embryonic brain in N2 culturing media (MEM/F12 media, HEPES, N2 supplement (LifeTechnologies)) for each experiment and the entire procedure was performed on ice. Tissue was dissected and collected into ice cold N2 media until dissociation step. For CD-1 postnatal tissue, mice were anaesthetized with isoflurane (Baxter) and perfused with cutting solution [87 mM NaCl, 2.5 mM KCl, 1.25 mM NaH_2_PO_4_, 26 mM NaHCO_3_, 75 mM Sucrose, 20 mM Glucose, 0.5 mM CaCl_2_^∗^H_2_O, 7 mM MgSO_4_^∗^7H_2_O] and tissue collected at P19 to P27. Ventral midbrain (SNc and VTA) dissection was done by 300 μm thick vibratome (Leica) coronal sections and SNc/VTA cut out with scalpel. Sectioning and cutting were completed in oxygenated (5% CO_2_/95% O_2_) cutting solution. For each experiment 2-3 mice were used (see Table S1) and 3-4 slices per brain. Entire procedure and solutions were kept cold and oxygenated. For transgenic *DAT1-Cre/tdTomato* mice, P28 and P56 non-anaesthetized mice were rapidly decapitated and their brains carefully removed and kept in ice-cold, artificial cerebrospinal fluid for dissection and dissociation (ACSF-D) [200 mM Sucrose, 2.6 mM KCl, 10 mM MgCl_2_, 0.5 mM CaCl_2_, 26 mM NaHCO_3_, 1.27 mM NaH_2_PO_4_ and 10 mM Dextrose] (equilibrated with 5% CO_2_/95% O_2_) (pH 7.3). Coronal, 400 μm thick slices were cut on a vibratome and transferred to ice-cold ACSF-D. The SNc and VTA were subsequently dissected out with a scalpel under the visual guidance of a fluorescent stereomicroscope.

Embryonic mouse, human, and postnatal CD-1 tissue pieces were processed similarly by dissociation using Papain Dissociation System (Worthington) following the manufacturer’s recommendations, adjusting incubation time based on tissue piece size, 25-45 min. Briefly, after papain incubation, glass pipettes of increasingly smaller tip diameter (fire-polished) were used to dissociate to single-cell suspension followed by a centrifugation through a BSA single step discontinuous density gradient. Then cells were filtered with 20 μm strainer (Partec CellTrics). Cells were pelleted, resuspended, and stored in N2 media with DNaseI until they were loaded into Fluidigm C1 chips for cell capture. *DAT1-Cre/tdTomato* midbrain tissue pieces were gently dissociated in 1 mL ACSF-D solution containing 1.1 mM EDTA, 10 mM L-Cysteine and 15U papain, activated for 15-30 min at 37°C. After dissociation the cell suspension was filtered (30 μm mesh) into 1mL of ACSF-D with 0.5% BSA and damaged cells stained with 0.1% Propidium Iodide (PI). Single cells positive for tdTomato and negative for PI were sorted on a FACS ARIA II (equipped with a 100 μm nozzle) directly into 3 μl of ice cold ACSF-D with 0.5% BSA in the cell collection chamber of a Fluidigm C1 chip to a final concentration 100-150 cells/μL. The collected cells were processed immediately after FACS on the Fluidigm C1 System according to the C1-STRT protocol (Zeisel et al., 2015). Briefly, C1-STRT (also called STRT-seq) is implemented on the Fluidigm C1 Single-Cell Auto Prep System. The protocol consists of cell capture, cell wash, imaging, cell lysis, reverse transcription and full-length cDNA PCR all performed in disposable microfluidic chips (Fluidigm C1 Single-Cell Auto Prep IFC for mRNA Seq, medium size, 10 μm – 17 μm). Following elution from the chip, the amplified cDNA is then tagmented using Tn5 transposase, purified and sequenced on the Illumina HiSeq platform. The following sections describe each step in brief. For detailed protocols, see (Islam et al., 2014).

#### Single cell isolation and cDNA synthesis

14 μl of cell suspension (approx. 800 cells/μl in N2 culturing media with DNaseI) was mixed with 7 μl C1 Suspension Reagent after filtering. Single-cells were then captured for 30 min at 4°C using the “Cell Load (1772x/1773x)” script. Bright-field imaging of every capturing site was performed on a Nikon TE2000E automated microscope using μManager (https://micro-manager.org/).

Immediately after the image acquisition, the chip was returned to the Fluidigm C1 System and the protocol for Lysis, RT and PCR were performed as previously described (Islam et al., 2014). After completion for the cDNA, the amplified cDNA was harvested with 13 μl Harvest Reagent and cDNA library quality was measured on an Agilent BioAnalyzer.

#### Preparation of sequencing library

The images of the capture sites were inspected and only capture site with single healthy cells were selected for library preparation. Cell barcoding and fragmentation, was performed in a single step using Tn5 DNA transposase (‘tagmentation’) as described previously. 1 μl Dynabeads MyOne Streptavidin C1 beads (Invitrogen) were resuspended in 20 μl Binding and Blocking buffer (10 mM Tris, 250 mM NaCl, 5 mM EDTA, 0.5% SDS) and added to each well. After 15 min incubation at room temperature, all wells were pooled, the beads washed once with 100 μl Washing buffer (10 mM Tris-150 mM NaCl, 0.02% Tween), once in 100 μl QIAGEN Qiaquick PB and then twice using 100 μl Washing buffer. Restriction was performed to cleave 3′ fragments: the beads were incubated in 100 μl restriction mix (1x NEB CutSmart, 0.4 U/μl PvuI-HF enzyme) for 1 hr at 37°C. Finally, the beads were washed three times with Washing buffer, then resuspended in 30 μl ddH_2_O and incubated for 10 min at 70°C to elute the DNA. AMPure beads XP (Beckman Coulter) were used at 1.8x volume and eluted in 30 μl to remove short fragments.

#### Illumina sequencing and bioinformatics pre-processing

The molar concentrations of the libraries was determined with KAPA Library Quant qPCR (Kapa Biosystems) and size distribution was evaluated after PCR (12cycles) using an Agilent BioAnalyzer. Sequencing was performed on an Illumina HiSeq 2000 with C1-P1-PCR2 as read 1 primer and C1-TN5-U as index read primer. Reads of 50 bp as well as 8 bp index reads corresponding to the cell-specific barcodes were generated. Reads were mapped using bowtie and processed as described previously (Zeisel et al., 2015), adding the more strict criteria for UMI counting: we removed all singletons (molecules supported by a single read).

#### hESC differentiation

Human ESC-derived dopaminergic neurons from H9 and HS401 cell lines were differentiated in a similar manner as Kriks et al. (2011) (Figure S7A). hESCs were dissociated into single cells by TrypLE Select (Thermo Fisher Scientific) and were plated on recombinant human laminin-111 (BioLamina) coated plates at a density of 600,000 cells per cm^2^ in Glasgow’s minimum essential medium (G-MEM) supplemented with 8% knockout serum replacement (KSR), 0.1 mM MEM nonessential amino acids, sodium pyruvate, and 0.1 mM 2-mercaptoethanol (all Thermo Fisher Scientific). Differentiation medium was gradually shifted to neurobasal medium with B27 supplement (Thermo Fisher Scientific) and 2 mM L-glutamine (Thermo Fisher Scientific) from day 5 to day 12. LDN193189 (100 nM; Stemgent) and SB431542 (10 μM; Tocris) were supplemented from day0, SHH C-24 (200 ng/mL; R&D) and fibroblast growth factor 8 (FGF8b; 100 ng/mL; R&D) were supplemented from day1, and CHIR99021 (3 μM; Sigma) was supplemented from day3. SB431542 and FGF8b were removed from the culture medium on day 7. Y-27632 (10 μM, Tocris) was supplemented for 24 hr after single cell dissociation. Cells were dissociated using TrypLE Select and replated on ornithine (50 μg/mL; Sigma) / fibronectin (2 μg/mL; Sigma) / laminin (3 μg/mL; Thermo Fisher Scientific)-coated plate at a density of 300,000 cells per cm^2^ in neurobasal medium supplemented with B27 supplement and 2 mM L-glutamine, GDNF (10 ng/mL; R&D), ascorbic acid (200 mM; Sigma), BDNF (20 ng/mL; R&D), 400 mM dbcAMP (0.5 mM; Sigma) and TGFβ3 (1 ng/mL; R&D) on day 12 and day 19. Culture medium was changed every 2-3 days. At day 0, 12/13, 18/19 and 35 cells from replicate wells were used for: qPCR analysis, 4% paraformaldehyde fixation for immunohistochemistry, and single-cell dissociation for RNA-seq using TrypLE Select and collecting in culturing media.

#### iPS derived dopaminergic neurons

iCell DopaNeurons were thawed and cultured in 48 well-plates previously coated with poly-L-ornithine (Sigma) and Laminin (Sigma). The plating density of the cells was between 120,000-160,000 cells/cm^2^ in four different experiments. The cells were incubated at 37°C, 5% CO2, for 5 and 21 days, with iCell DopaNeurons Maintenance Medium (DNM-301121001) complemented with iCell DopaNeurons Medium Supplement (DNM-301031001) and iCell Nervous System Supplement (NSS-301031001) provided by Cellular Dynamics Int. At two different time points, day 5 and day 21, some wells were fixed with 4% paraformaldehyde and used for immunohistochemistry and the rest of the wells were used for single-cell RNA-seq analysis; after trypsinization with TrypLE Select, cell resuspension was kept in the same maintenance medium with albumin from bovine serum.

#### Reverse transcription and qPCR

Total RNA was extracted using an RNeasy Mini kit (QIAGEN). 200-500 ng of total RNA was used for reverse transcription by a Super Script II First-Strand Synthesis System with random primer (Invitrogen). qPCR was performed by using a StepOne detection system (Applied Biosystems). Data analysis is based on the ΔΔC_T_ method with normalization of the raw data to *GAPDH* genes. Primer sequences are shown in Table S4.

#### Immunohistochemical analysis

After mouse embryos (E12.5-E18.5) or postnatal brains were fixed in 4% paraformaldehyde for at least 6 hr, they were processed in 30% sucrose and embedded in OCT (Tissue Tek) then cryostat sectioned at 16μm. Human fetal tissue for cryosectioning was immersion-fixed overnight in 4% paraformaldehyde at 4°C, then cryoprotected in sucrose before embedding in OCT compound, then 14 μm sections were cut using a Leica cryostat. According to manufacturer’s antibody recommendations, some sections were treated for antigen retrieval by microwave boiling (S1699, DAKO). Sections were washed in PBS and incubated in blocking solution, PBTA (PBS, 5% normal donkey serum (Jackson ImmunoResearch), 1% BSA, 0.2% Triton X-100). Sections were incubated overnight at 4°C in primary antibody. Slides were washed and then incubated for 1-2 hr at room temperature with corresponding Alexa Fluor fluorophore-conjugated secondary antibodies and nuclei counterstained using DAPI.

For hiPSC and hESC in vitro cultures, after incubation for 1 hr in blocking solution, the cells were incubated overnight at 4°C with a series of combinations of different primary antibodies. After removal of the primary antibodies, wells were washed with PBS and incubated for 1 hr with corresponding secondary antibodies and nuclei counterstained.

Image capture was done with Zeiss LSM700, Zeiss LSM780 or Olympus FV1000 at CLICK facility. Images were processed with Adobe Photoshop and Illustrator.

#### In situ hybridization

For in situ hybridization, embryos at E12.5 and E13.5 were fixed (4% paraformaldehyde, 4°C) overnight then cryopreserved in 30% sucrose and embedded in OCT for sectioning, 14 μm. Sequences for primers for the production of mouse antisense RNA probes were obtained from Allen Institute for Brain Science, and are given in the Key Resources Table. In situ hybridization was performed using Digoxigenin-labeled probes and detection with alkaline phosphatase-conjugated antibody, as described previously (Conlon and Herrmann, 1993). Briefly, 4% paraformaldehyde preserved and cryo-embedded embryos were sectioned coronally at 14 μm. Probes were synthesized with DIG RNA and SP6 polymerase and hybridized to sections overnight at 70°C. After thorough washing and blocking, anti-Digoxigenin-AP Fab fragments were incubated on slides overnight at 4°C. After washing, slides were developed using NBT/BCIP solution. Where indicated, in situ images were resourced from Allen Institute for Brain Science: Allen Mouse Brain Atlas (Lein et al., 2007) and Allen Developing Mouse Brain Atlas. Available from: http://mouse.brain-map.org and http://developingmouse.brain-map.org, respectively.

#### Single molecule RNA FISH

RNA smFISH was carried out as previously described (Zeisel et al., 2015) with minor modifications. 10 μm thick sections of E11.5, E13.5 and 15.5 embryos were mounted on the same cover glass, post-fixed with 4% paraformaldehyde for 10 min at room temperature and permeabilized with methanol. In order to perform sequential hybridization, the cover glass was mounted on a custom-made imaging chamber. After assembly, the chamber was incubated for 10 min at 70°C in Tris-EDTA (pH 8.0). The sections were then washed twice with SSC 2X and incubated with hybridization buffer containing 250 nM fluorescent label probes (LGC Biosearch Technologies) for 4 hr at 38.5°C. After four 20% formamide-SSC 2X washes, the slides were counterstained with Hoechst, washed with SSC2X and imaged in Slow Fade mounting medium (Thermo Fisher Scientific). Image stacks (0.3 μm distance) were acquired using a Nikon Ti-E motorized inverted microscope. After imaging, the hybridized probes were stripped in 65% formamide and washed three times in 2X SSC. The sections were re-imaged in order to confirm that no probe signal was detectable. The hybridization-imaging-stripping procedure was then repeated two more times. Sequences of probes for all genes are found in Table S3.

The images were analyzed using the Python numpy, scipy.ndimage (Jones et al., 2014) and scikit-image (van der Walt et al., 2014) libraries. Briefly, after background removal using a large kernel Gaussian filter, a Laplacian-of-Gaussian was used to enhance the RNA dots. Background objects significantly larger (60 times the area) than the smFISH dots were removed after image thresholding. The images were then stitched, aligned, and pseudocolored in Fiji (Schindelin et al., 2012). To facilitate visualization the size of dots was increased using a dilation filter.

### Quantification and Statistical Analysis

#### Quality control

Taking in to consideration the distribution of molecules per cell in each dataset we set both a lower and an upper threshold (2000-26000 molecules/cell for mouse embryo cells, 2000-30000 molecules/cell for adult mouse cells, 1200-24000 molecules/cell for the human embryo cells and 2200-13000 molecules/cell for the hPSCs) so that we could eliminate cells outside of this range with the aim of removing data from both broken cells and doublets that might have gone undetected despite the imaging. We further decided to exclude cells that had inconsistent assignment with different BackSPIN parameters and low molecule count. For the hiPSCs and the hESCs this last step of filtering was not performed since it may introduce bias when estimating the abundance of different kind of cells present in the culture. Genes that were detected at less than 4 molecules in the whole datasets were eliminated. After these quality control procedures, we were left with: 1907 cells for the mouse embryo dataset with detected transcripts between 929 and 6063 (median 2831); 1977 cells from the human embryos with detected transcripts between 717 and 5459 (median 2292); 279 cells from the adult mouse with detected transcripts between 1445 and 6498 (median 4033); 337 cells from the hiPSC culture with detected transcripts between 983 and 4411 (median 2284) and 1715 cells from the hESC culture with detected transcripts between 959 and 7110 (median 3635).

#### Clustering and cluster analysis

Cells were clustered using a version of the BackSPIN algorithm optimized for this dataset. We used a Bayesian generalized linear model (GLM) to assign every gene to one or more cell populations, as previously described (Zeisel et al., 2015). We used BackSPIN, an iterative clustering approach that alternates feature selection and clustering. Briefly, we first selected genes with the highest variation and then we ran BackSPIN with low splitting depth to reveal major splits in the data. (e.g., vascular, neurons, cycling cells). We then undertook a further feature selection on each of these high level clusters and we reclustered them to resolve finer differences. In this way, we avoided the initial feature selection imposing a subspace that would mask biologically relevant differences.

The feature selection procedure is based on the largest difference between the observed coefficient of variation (CV) and the predicted CV (estimated by a non-linear noise model learned from the data) See Figure S1C. In particular, Support Vector Regression (SVR, Smola and Vapnik, 1997) was used for this purpose (scikit-learn python implementation, default parameters with gamma = 0.06; Pedregosa et al., 2011). Importantly, after defining two clusters A and B, we used a procedure to avoid considering genes peculiar to B when reclustering A and vice versa. This is undesirable because it could result in calling spurious clusters as a result of carryover or occasional contamination.

The procedure was performed as follows: (1) Starting with the set of genes assigned by BackSPIN to B (2) We refined it by defining a core set of genes highly correlated (3) We then searched among all the genes the ones that were highly correlated with most of the core genes, (4) and excluded all the genes so identified when reclustering A.

The steps of the BackSPIN algorithm (Zeisel et al., 2015) are described in detail below.

For the analysis of the human dataset we proceeded as follows. (1) We performed a first feature selection and then ran BackSPIN. At this level cell-cycle genes had an important effect on clustering, making it more difficult to separate cell types. Therefore, we removed cell-cycle genes with the procedure described above and analyzed cell-cycle state separately (see below). (2) We performed a feature selection of 2000 genes and ran BackSPIN clustering on the whole dataset. Clustering revealed a major split between non-neural and neural cells. (3) We then proceeded with the clustering of the neuronal cells finding a major separation between ventricular zone cells and neuroblast/neurons. (4) Each of these subclusters were then clustered at high depth (numLevels = 7, runs_iters = 12, runs_step = 0.1, stop_const = 1.1). The clustering of the mouse was performed following the same procedure.

To avoid unreliable splits, we manually inspected the clustered matrix and rejected those splits that did not show obvious gene expression differences, and reconstituted the cluster at the previous level. This adjustment thus resulted in more conservative clusters in some cases, but never resulted in the creation of new clusters not supported by the algorithm.

To visualize the high dimensional data in two-dimensional space and to validate our clustering result we used t-stochastic neighbor embedding (t-SNE; der Maaten and Hinton, 2008). As a fair comparison, we calculated t-SNE projection using the same 2000 genes that were used as the initial input in the BackSPIN algorithm.

Adult mouse dopaminergic neuronal data were clustered together with dopaminergic neurons from the embryonic dataset to allow clusters including both the embryonic and adult cells (however, the difference was clear and BackSPIN separated these two datasets with great fidelity). In this analysis, similar to what we had described before for the iterative BackSPIN we filtered away genes that were significantly higher in other cell types in the adult VM (p < 0.005) and selected fewer genes (100) for the BackSPIN clustering.

Similarities between clusters within a species were summarized using a Pearson’s correlation coefficient calculated on the binarized matrix (Figures 1C and 1D). For the calculation all genes that were significantly expressed over baseline levels in at least one of the clusters were used, but if a gene was detected at less than 1 molecule per cell in all the clusters, it was excluded from the calculation. Correlation matrices were then sorted by SPIN (Tsafrir et al., 2005) for easier visualization.

#### BackSPIN Algorithm

BackSPIN is a two-way clustering algorithm crafted to handle large datasets and developed taking into account intrinsic features of single-cell RNA-seq experiments. BackSPIN uses SPIN (Sorting Points Into Neighborhoods, Tsafrir et al., 2005) as the engine for sorting correlation-based distance matrices in a one-dimensional order. Briefly, SPIN sorts a distance matrix by iteratively permuting rows (or columns) while maximizing an objective function that penalizes the separation of similar rows (or columns).

BackSPIN extends SPIN by implementing a splitting procedure to divide the sorted data matrix into sub-matrices that finally yield coupled clusters of cells and genes.

Let *A* be a matrix *(m x n)* typically containing gene expression where columns represent cells and rows genes. The aim is to obtain clusters of cells (columns) with their corresponding overexpressed/enriched genes. The algorithm is composed of two main functions:

(1) A SPIN-sorting of a correlation matrix: returning a permutation (one dimensional order) that optimizes the SPIN objective function (Tsafrir et al., 2005).

(2) A splitting step. Allowing for *C* to be the sorted correlation matrix from the previous step, it finds the optimal splitting point *x*_*s*_ such that it maximizes an objective function defined as:

(1) $$f\left(x\right)=\frac{\sum_{i=0}^{x}\sum_{j=0}^{x}C_{i,j}+\sum_{i=x+1}^{n}\sum_{j=x+1}^{n}C_{i,j}}{x^{2}+{\left(n-x\right)}^{2}}\;\text{with}\;i\neq j\Rightarrow x_{s}=\begin{array}{c} \arg\;\max\;f\left(x\right)\\ x \end{array}$$

The steps of the algorithm are as follows:

STEP1: Sort the samples (cells) correlation matrix *C* iterating SPIN with gradually decreasing width parameter.

STEP2: Find *x*_*s*_ and divide the matrix *A* in two sub-matrixes *K* and *L*. For each gene, the two sub-matrixes are considered in the orientation that maximizes the distance of their center of mass from the point of split. Then, the center of mass over the parent matrix is calculated and the gene assigned to the cluster of cells this falls in.

STEP3: If *f(x)* is greater than a threshold (stopping condition) repeat STEP 1 and 2 for K and L. When *f(x)* is smaller than a threshold or max splitting depth is reached, then stop splitting.

STEP4: SPIN-sort the samples and construct a features correlation matrix of every sub-matrix generated.

The rationale for using the center-of-mass for assigning genes to sub-matrices is as follows. Once the matrix A has been split in two sub-matrices K and L, the goal is to assign each gene to either K or L. A statistical test could be used to determine, for example, if the gene is expressed at a higher level in K than in L, or vice versa. However, it may be that the gene is truly expressed only in a small subset of the cells in K, whereas it has a broad low-level expression in all cells in L. Thus the gene is more relevant for subsequent clustering of K than of L. But since both K and L are ordered by SPIN (the first step in each BackSPIN iteration), we can detect such expression in a small (and correlated) subset of cells by using the center of mass. In a sense, the center of mass lets BackSPIN peek into the future and detect clusters of cells that will only be discovered in later iterations. The key feature of center-of-mass is that it exploits the correlation-based ordering of cells, which provides a signal that a gene is relevant for defining subsets of cells in future iterations of the algorithm. In contrast a two-group statistical test will simply reveal which group has a higher expression, not which group has a more structured and informative expression of the gene.

#### Marker discovery and binarization by Bayesian regression

We used a Bayesian generalized linear model (GLM) to assign every gene to one or more cell populations. The GLM models the measured gene expression of a cell as realizations of a Negative Binomial probability distribution whose mean is determined by a linear combination of K  predictors ${\text{x}}_{\text{i}}$ with coefficient ${\text{β}}_{\text{i}}$.

$$\text{μ}=\sum_{\text{k}=1}^{\text{K}}{\text{β}}_{\text{k}}{\text{x}}_{\text{k}}\;\text{k}\in \left[1,\text{K}\right]$$

For each cell, the outcome and predictors are known and the aim is to determine the posterior probability distributions of the coefficients.

As predictors, we use a continuous *Baseline* predictor and a categorical *Cell Type* predictor. The *Baseline* predictor value is the cell’s molecule count normalized to the average molecule count of all cells and takes account of the fact that we expect every gene to have a baseline expression proportional to the total number of expressed molecules within a particular cell. While the *Cell Type* predictor is set to 1 for the cluster BackSPIN assignation of the cell, and 0 for the other classes. From the definition of the model it follows that the coefficient ${\text{β}}_{\text{k}}$ for a *Cell Type* predictor ${\text{x}}_{\text{k}}$ can be interpreted as the additional number of molecules of a particular gene that are present as a result of the cell being of cell type *k*. A more detailed description of the model, including explanation of the prior probabilities used for the fitting as well as the full source code of the model, is provided elsewhere (Zeisel et al., 2015). The Stan (http://mc-stan.org) source is copied below for completeness:

data {

 int < lower = 0 > N; # number of outcomes

 int < lower = 0 > K; # number of predictors

 matrix < lower = 0 > [N,K] x; # predictor matrix

 int y[N]; # outcomes

}

parameters {

 vector < lower = 1 > [K] beta; # coefficients

 real < lower = 0.001 > r; # overdispersion

}

model {

 vector < lower = 0.001 > [N] mu;

 vector < lower = 1.001 > [N] rv;

 # priors

 r ∼cauchy(0, 1);

 beta ∼pareto(1, 1.5);

 # vectorize the overdispersion

 for (n in 1:N) {

 rv[n] < - square(r + 1) - 1;

 }

 # regression

 mu < - x ^∗^ (beta - 1) + 0.001;

 y ∼neg_binomial(mu ./ rv, 1 / rv[1]);

}

To determine which genes are higher than basal expression in each population we compared the posterior probability distributions of the *Baseline* coefficient and the *Cell Type* coefficient. A gene was considered as marking a cell population if (1) its cell-type-specific coefficient exceeded the Baseline coefficient with 99.8% (95% for the mouse adult) posterior probability, and (2) the median of its posterior distribution did not fall below a threshold θ set to 35% of the median posterior probability of the highest expressing group, and (3) the median of the highest-expressing cell type was greater than 0.4. For every gene this corresponds to a binary pattern (0 if the conditions are not met and 1 if they are), and genes can therefore be grouped according to their binarized expression patterns.

We use those binarized patterns to call transcription factor specificity. Our definition of a transcription factor gene was based of annotations provided by the merged annotation of PANTHER GO (Mi et al., 2013) and FANTOM5 (Okazaki et al., 2002), this list was further curated and missing genes and occasional misannotations corrected.

#### Cross-species comparison

First we used the Bayesian model described above to obtain for every gene the Maximum a posteriori (MAP) estimate of the cell-type specific mean parameter. To make the gene expression profiles of mouse and human cell types comparable we used the homology correspondences provided by the Homologene database (Wheeler et al., 2007). We considered only the biunivocal correspondences, while the one-to-many correspondences (constituting a minority of the database) were discarded.

With the datasets in the same feature space, it is possible to calculate a similarity measure between cell types of different species. We found that, in this context, a naive correlation coefficient calculated over the full transcriptome was biased to assign high similarity because of confounding factors such as size or number of expressed genes. We therefore further filtered the feature space to reduce it to the genes that were expressed with some significant variation across the cell types. This was defined using the following filter: select genes that are (1) significantly expressed (according to the Bayesian condition described above) over baseline in at least one cell type in both species but (2) in less than 6 cell types in at least one of the species and (3) with a maximal expression that was bigger than 1.5 molecules per cell and the smaller than 0.25 in either species. 1405 genes passed this filter and were used for the calculation of the correlation coefficient.

Two cell types were considered to be correspondent when they satisfied the following condition:

$$S_{n,m}\geq S_{n,j}\;\forall j\in \left\{\;Mm\;celltypes\;\right\}\wedge S_{n,m}\geq S_{i,m}\;\forall i\in \left\{\;Hs\;celltypes\;\right\}$$

Where S_m,n_ is the correlation between a human cell type m and mouse cell type n.

For the time comparison, we estimated the time when half of all the cells sampled had been observed. We estimated this quantity by first fitting a continuous smooth function to the time points and then finding the point where the area under the curve was half of the total.

#### Proliferation index

We developed a cell-cycle scoring approach that uses expression data to compute an index for every cell that scores the cell according to its expression of cell-cycle genes. In brief, our approach proceeded through four steps. (A) We reduced dimensionality of the dataset to the cell-cycle relevant genes. (B) In this subspace we performed, as a first approximation, a simple K-means clustering to separate non cycling from cycling cells and (C) we used this clustering as a reference to learn a function that takes the gene expression as the input and returns a cell-cycle score as an output. (D) We used this function to calculate a score for each single cell.

We started by selecting a wide selection of genes related to cell-cycle and proliferation. We used the PANTHER GO database and selected all the genes that were described by one of the following terms: *DNA metabolic process, DNA replication, mitosis, regulation of cell cycle, cell cycle, cytokinesis, histone, DNA-directed DNA polymerase, DNA polymerase processivity factor, centromere DNA-binding protein*. We restricted our features to those genes. Genes that were detected at less than 10 molecules in the dataset were removed. We calculated the pairwise correlation coefficient matrix, and selected the genes that were strongly correlated (99^th^ percentile of the matrix) with at least 12 other genes. The genes passing the filters described above were used for clustering cells using K-means (Python scikit-learn implementation, on log-centered data, default parameters) with the rationale that the main axis of variation expected would span across dividing and non-dividing cells. Then a linear regression model with L1-norm regularization was fitted that used a learning function which took expression data of a cell and categorized into two classes, 1 when a cell belongs to the cycling cluster and 0 when it did not. Importantly, to avoid both overfitting the score on the first approximation clusters and also to obtain a more generalizable model, we used a strong regularization (5 times the one determined by cross-validation; alpha = 0.01).

This procedure was used for both the mouse and human embryonic dataset. The function learnt on the human embryonic dataset was also used to determine the proliferation index of the hPSCs.

#### Pseudotime analysis

We analyzed the variation of gene expression over time in the spirit of the recent work by Magwene et al. (2003) and Trapnell et al. (2014). However we used a different algorithmic approach exploiting the mathematical properties of principal curve, which can be considered a nonlinear generalization of a linear principal component (Hastie and Stuetzle, 1989). In brief, the principal curve is a smooth one-dimensional curve that passes through the middle of a data cloud in n-dimension space. Therefore, the use of principal curve does not require reducing dimensionality down to 2 dimensions or building a graph. Noticeably, using principal curves we did not have to artificially force our time path to pass through every cell point. Our approach consisted of the following steps: (1) Finding a subspace that was time relevant. First, we selected the top 5000 genes using the CV-mean relation and then we tested which of those genes varied significantly in time (embryonic day of sampling). We used an approach that conceptually corresponds to performing an ANOVA for over-dispersed data: we performed a likelihood ratio test comparing a GLM with a Negative binomial link function and time (E-day) as a categorical predictor against the *null* model that does not take time in account. Only significant genes (FDR < 0.01) were considered for the following steps. We also excluded genes that were significantly expressed in other cell types but not in the cell type of interest. (2) We projected data using PCA and we selected the principal components that had a SD bigger than 0.25. Finally, we calculated the principal curve passing through the points in this multidimensional space (using the R package princurve; Weingessel and Weingessel, 2015). At this point, the curve could be projected back into the original gene space and by so doing offer insight on the variation of genes in time. However, this can be done only for the original time-relevant features taken in to account for the PCA calculation. So to make it possible to generalize it for every gene we projected every cell on the curve and assigned to every cell a principal time, corresponding to the length of the arch from the beginning of the curve. In this way, every gene could be represented as a function of pseudotime. (3) We then fitted a curve pseudotime-expression using SVR (parameters where chosen by crossvalidation using a stratified KFold procedure). Before fitting, the normalized molecule count data were corrected for missing values using a Lasso regression approach described elsewhere (Satija et al., 2015). (4) The pseudotime dependent profile for every gene was clustered using affinity propagation. Genes with R-squared smaller than 0.35 were considered not significant and not included in the clustering. This procedure resulted in a set of prototypical dynamics. The top genes ranked by the coefficient of determination (R-squared) of the prediction presented are shown in Figure S6A.

#### Cell scoring using machine learning

For the analysis on in vitro-derived cells (Figure 7), our goal was to score the molecular identity of each cell relative to in vivo-defined cell types. For this purpose, we first calculated cell-type prototypes representing the idealized in vivo expression patterns. These prototypes were then used as references to which individual cells could be compared, resulting in a probabilistic similarity score.

A logistic regression with L2-norm regularization and a multinomial learning approach (implemented by the scikit-learn function LogisticRegression; (Pedregosa et al., 2011)) was trained using the log-transformed max-normalized data. As a training data for the classifier, we used the human embryonic dataset, including, as a relevant outgroup, the early hESC data (day 0 of the *in-vitro* differentiation experiment). We trained the model to learn more general cell-type prototypes, rather than restrict itself to the 25 clusters. Prototypes consisted of either a single cluster (for example Serotonergic prototype is learnt on hSert) or by several subclasses that are biologically related (the Dopaminergic prototype is learnt from hDA0, hDA1 and hDA2). The composition of the prototypes consisting of more cell clusters were: Embryonic stem cells (eES) consisting of eSCa, eSCb and eSCc. Floor plate progenitors (ProgFP) consisting of hProgFPM, hProgFPL and hProgM. Radial glia (Rgl) consisting of hRgl1, hRgl2, hRgl2b, hRgl2c and hRgl3. GABAergic lineage consisting of hNbML5, hNbGaba and hGaba.

To train the model, the top 4500 most variable genes were chosen by CV-mean relation as above, and refined as follows: (1) To avoid learning cell culture specific features instead of ES features we discarded genes that had minimal variability in the whole hESC differentiation experiment when compared to the combined dataset (2) To eliminate genes whose variation is orthogonal to cell-types and produce a more general model the gene list was further reduced to half. We did this by choosing the top genes ranked by three heuristics for cell-type specificity (fold-increase, fold-increase^∗^fraction-positive, fold-increase^∗^ fraction-positive^0.5^) (Marques et al., 2016).

To choose the adequate regularization parameter for the logistic regression, the classifier accuracy and sum of regression coefficients were plotted against progressively less stringent regularization parameters and inspected (Figures S7H and S7I). The value of regularization (C = 0.01) was chosen as it corresponds to the point that has maximum accuracy before the plateau is reached. The average accuracy was estimated by a cross-validation procedure: for 35 iterations the dataset was randomly split (following a stratified k-fold approach) the bigger part (85% of the dataset) was used to train the classifier and the remaining 15% was used as a test set to compute the accuracy score (fraction of correctly predicted sample over the total).

Finally, the model was used to predict the probabilities of each cell belonging to each one of the prototype classes, the predicted probability of each class is calculated using the softmax function (implemented by the predict_proba method of the logistic regression model, scikit-learn).

Data were plotted on a “wheel plot” polygon by calculating the position of each cell as a linear combination weighted by the probabilities emitted by the model. That is: let *p*_*i*_ be the probability of a cell belonging to prototype class *i* and let ${\hat{u}}_{i}$ be the unitary vector of origin the center of the polygon and directed toward the i^th^ vertex of the polygon, the position $\vec{xi}$ is, then, given by: $\vec{xi}=\sum p_{i}{\hat{u}}_{i}$. To make this representation clearer and more informative, the order of the vertices was chosen to minimize the number of cells in the central area of the plot.

### Data and Software Availability

#### Data Resources

The accession number for the raw data reported in this paper is GEO: GSE76381.

Cell-scoring command-line tool and ipython notebooks showing key steps of the analysis are available for download on Github at: http://github.com/linnarsson-lab/ipynb-lamanno2016. BackSPIN algorithm is available on Github: https://github.com/linnarsson-lab/BackSPIN.

### Additional Resources

Plots for any gene can be visualized at http://linnarssonlab.org/ventralmidbrain.

## Author Contributions

G.L.M. performed single-cell RNA-seq experiments and analyses, developed computational tools, made figures, and wrote the paper; D.G. dissected mouse brain tissues, performed immunohistochemistry and in situ hybridization, analyzed data, made figures, and wrote the paper; S.C. and L.E.B. performed RNA smFISH and image analysis; C.S. and K.N. performed hPSC experiments; S.R.W.S. dissected human midbrain tissue; A.Z. performed RNA-seq experiments, developed computational tools, and discussed the draft paper; E.M.T. performed analyses; J.R. performed DAT1-tdTomato RNA-seq experiments; J.C.V. performed immunohistochemistry; P.L. performed bioinformatics; R.A.B. provided human fetal tissues and critically reviewed the manuscript; E.A. and S.L. conceived of and supervised the project, analyzed data, made figures, and wrote the paper; and all authors read and commented on the manuscript.

## Supplemental Information

Document S1. Tables S1, S3, and S4

Table S2. Binarized Genes Across Cell Types, Related to Figure 1
