# Supplementary material for: Molecular Diversity of Midbrain Development in Mouse, Human, and Stem Cells
Source: Cell. 2016 Oct 6;167(2):566–580.e19. doi: 10.1016/j.cell.2016.09.027 (PMC5055122; doi:10.1016/j.cell.2016.09.027)
Supplement: Document S1. Tables S1, S3, and S4 [file mmc1.pdf]

Cell, Volume *167*

Supplemental Information

Molecular Diversity of Midbrain Development in Mouse, Human, and Stem Cells

Gioele La Manno, Daniel Gyllborg, Simone Codeluppi, Kaneyasu Nishimura, Carmen Salto, Amit Zeisel, Lars E. Borm, Simon R.W. Stott, Enrique M. Toledo, J. Carlos Villaescusa, Peter Lönnerberg, Jesper Ryge, Roger A. Barker, Ernest Arenas, and Sten Linnarsson

**Table S1. Related to Figure 1. Tissue used per experiment**						
Mouse (embryonic) CD-1						
Embryonic Age (E)	Embryos used per experiment (each cell is a separate day; number represents litter mates collected from one mother)					Total (n=)
11.5	12	15	12	11	13	5
12.5	11	15	13	14		4
13.5	15	11	14			3
14.5	7	16	12	13	12	5
15.5	15	14	10			3
18.5	6	10				2
Total embryos collected					271	
Total embryonic experiments						n=22
Mouse (postnatal) CD-1						
Age (days)	Brains used per experiment (each cell is a separate day)					Total (n=)
P19	2					1
P20	2	3				2
P21	2	3	3			3
P23	2	3				2
P24	3	3	3			3
P25	3					1
P26	3	3				2
Mouse (postnatal) Dat1-Cre/tdTomato						
Age (days)	Brains used per experiment (each cell is a separate day)					
P26	1					1
P28	1					1
P56	1	1	1			3
Total postnatal experiments						n=19
Human (fetal)						
Age (weeks)	Each cell is a separate day. 1+1 means that a second experiment is completed 24h after first with the other half of same tissue					Total fetuses
6	1					1
7	1	1				2
7.5	1					1
8	1+1					1
9	1+1	1+1				2
10	1	1+1				2
11	1+1					1
Total						n=10

hPSC cultures						
	Each cell is a separate day. Number indicates number of separate biological replicates (different cultures) run on that day, samples not pooled.					
H9						
Day 0	2					
Day 12	4					
Day 18	2					
Day 35	4					
HS401						
Day 0	2					
Day 12	4					
Day 17	2					
Day 35	4					
iPS (iCell DopaNeurons)						
Day 5 (47)	4					
Day 21 (63)	4	4				

Table S3. Related to Figure 3. Probe sequences (5’ -> 3’) for single molecule RNA FISH

Probe	Ednrb	Cd36	Foxj1
1	agtaagttaagtttgcgcgc	actctgaaaggatcagcact	ccagaggaaggttctggaag
2	tctgaaaacacactagcccg	acagcaatgtatgagctgtt	ctccgaacacgaatgtgagg
3	acaggacacttaagtcagct	ccggttctactaattcatgc	gaacactcacttccattctg
4	ttttcagtctggagaccaac	ccaggtacaatcacagtgtt	cagactccagttacacagtc
5	cgattgcatgttacagcttg	gcagaatcaagggagagcac	gtcaacatccacaggctgag
6	ttagtcccgagaagtgacag	taacagctccagcaatgagc	cttggcgttgagaatggaga
7	aggaacgcatcagactggag	tctcaataagcatgtctccg	gagatgtgcacgacgatgtg
8	caatatttcgttggcacgga	acaacttccctttgattgt	ataggagtagggtggcttca
9	acacgaggcacgacacaatc	tttgaaagcagtggttcctt	catggccatgcagatgagag
10	agatgattcttagcagcgtg	aactgtctgtacacagtggt	cgacagagtgatcttggtgg
11	gcgatcaagatattgggacc	atctgggttttgcacatcaa	ccgtgatccacttgtagatg
12	tgatgatgtgcagtaggtct	gattttgctgctgttctttg	catggcggaagtagcagaag
13	cgagcaacttgtaggtgtta	atgtgtaaggacctctttgt	aggcactttgatgaagcact
14	gagcacaaagactcagcact	gagttatattttccttggct	aaaggcagggtggatgtgga
15	ccaagaagcaacagctcgat	aaagacacagtgtggtcctc	tcaaactcctgaagcagctg
16	gaaccccaattcctttaatt	gtgaaggctcaaagatggct	cagcagtaaggtgctgggag
17	aatttctactgctgtccatt	cacaccaactgtggtactta	ttacctttgagtggttccag
18	cacagagaccacccaaatta	ccatctacagtgtcattgta	aaagatggcctcccagtcaa
19	ctttgtagtccgacgtaatc	cttatgttatcctttccatt	ggactgtaagatccacatct
20	aggctgttttctgaaaggga	tgccattaatcatgtcgcaa	gtagcagggcagttgatgtg
21	ggctgtcttgtaaaactgca	ccgaacacagcgtagataga	atcaaagtccaggctgtcag
22	aagactgcagtgatggctag	ggattcctttaaggtcgatt	gaaggatgtggccaagaagg
23	caaagcaatctgcataccgc	ggcattggctggaagaacaa	ttcaaagatgggttctgggg
24	tcgtctctgctttaagtgat	aatggttgtctggattctgg	ttttacaagaaggcacccac
25	aaagctcagaagctcacacc	tctggacttgcatgtaggaa	ttgagcactgtccggagatg
26	gttgataccaatgtagtcca	gtaagccttcaataggttct	ggcttggatatgaggacatc
27	tgcaggagttcaaagaagcc	cctatgctcatcttcatttg	aaacactgtgtgtatggggg
28	tgctcaccaaatacagagcg	gttatgggttccacatctaa	tgaggcgaagctttggtgag
29	accagcagcacaaacatgac	accaatatgttgacctgcag	ccagagaagcagcagaagtg
30	aggactgcttttcctcaaac	cttctatttttctagctggc	ggaactttctctgggtagag
31	tggaccggaagttgtcatat	catttagccacagtataggt	taatgagcgtttgttgtacc
32	ccttcaagacgagctgtatt	atgccaaggagcttgatttt	cctcctgggaattaatttgt
33	agaatgggaatatttcccca	caacaaacatcaccactcca	cgggcttagagaccatttcg
34	atgacgtggcagtgacgaac	ttcttggatttgcaagcaca	aggtgatggaccagggaaag
35	acaaggttaaagctctcggg	gctcatccactacttatttt	aagtcaggctggaaggtttg
36	cattttcaacatgtggcctg	gatgtagccagtgtatatgt	catcttcctgatgctcttag
37	ccgtttgggtttattgtatt	ttttgtagatcggctttacc	cccagttaagtcttagctat
38	tgttcttggtttttacctc	agtccactcagttgcaaatg	tagtatagacaggggcctag
39	catgtgtcacgtggattcag	cctccagtgagggaaaacaa	aggggtcaaggtggagcaag
40	actgtgggtgactcctaaag	agttgcaagccaaatttgct	cacgtggagggtagattcag
41	aattgaagatctggggcgtc	gttatacttatgccatgtgc	
42	actactggggagccaataac	cagtggctcacatgtgaatt	
43	attattgcccatgaagaggg	cttgctgtagccaagaactc	
44	tgccatttcataatttggca	tgcagcatggaacatgacga	
45	gacctctaacatccttgtta	ttgcttttgtaggattccat	
46	tttggctgttgcacattatc	aaaccagtcctttttttggg	
47	atgttaggtacaacacaccc	acaacctatcactgtgtctt	
48	ttagcaacgacacaagctgc	taatgacatcgtttcccaca	

Probe	Msx2	Rfx4	Slc6a11
1	ctcaagcgaagactccaact	agtaatccagtcgggcaaag	cagctacgctcaacacgaac
2	ggagaagccatgactttctg	gagacatctttcaatccagc	tttgtagcacaggtagggaa
3	gaaaacaagtcaccgccttt	ccagagatgtgtgactggaa	caccacgtaaggaatcagga
4	cttcttgtcggacatgagcg	gcaatctcatagttttcctc	cgtttccaggaagaagacgg
5	ctcgaagggcttgacgagag	cttctcacagaaatccaggt	gccaggatgatgatgtagta
6	tcttcggaattttccgactt	aaagctggcagcattgacag	ggtgaagcaattgcttaggt
7	ttggtcttgtgtttcctcag	aaactgctgccttatgatct	tggaactccacacatttctc
8	gggatgtggtgaagggtgtg	aatggtactttgactgtcct	tgtagttgctgaagttcagc
9	ttgcgctccaaggctagaag	ctggcagatttaggtctttg	tcaatgccatcggatatagc
10	atggacaggtactgtttctg	tctgacagtgtgttctgtac	caggagacataatgccagtt
11	tctggaaccagattttgacc	gaacctcatcaaagttggct	tgcagaagtagcagatggtc
12	ttgcagtcttttcgccttag	ccttgccaaaagtgcagaag	ctttccagtcgacttagtac
13	attttcagcttttccagttc	gtagaggatggagtcacaca	aggttgcagtgacatacacg
14	aagggcagactgaagcctga	tccatggtatacagagtctg	gaggtcagggtacagataga
15	ttgcaggggtgagttgatag	aactcctggtacaactggat	ctgtttagacagcagagcat
16	taggatgcgccgtatatgga	atccagccattctatgtaag	ctgagaagatggcaaaccca
17	gagcacaggtctatggaagg	ctttacaacgcatcggtcta	cttggggtaggcgatgaaag
18	cgtggcatagagtccaacag	aagcaagaccacatcagcag	acacaaactgactgtccagg
19	tggtacatgccatatccaac	aggtgaaaagacccgaagct	ggaagaccttagggtacatg
20	atctggtcttccttaggata	ttctagcaagtagagcacgt	tggcggcgtatgagtcaaaa
21	cgcattcaggagcagagttg	gcctctgtcaagataatctc	acaaagagcaagcacatgcc
22	tgtcgcttagggtgacaatg	cacagatgtggcagactttg	ccgatgcagacacactcaaa
23	gtgcctctcacctaaataac	tagcgaccacgtatatgact	attgtcatagaaccggttgc
24	tgagaggaaagggggcattt	tagttgttgggagttctcag	cggtaaccaatcatatcctc
25	gaagtgacatcctgtatgga	tcctgtggtaaggggaatag	ccacttgatgagtgacagtg
26	ccttcacttgcaagaatctg	tggggctattgaaagggtac	tgtacttgagcggtttgtac
27	attctcatcctcttttcatc	tcaaacaaggttccatcctg	ccaagcaggatatgtgtaca
28	ttaccctgagtaactctcag	ggtgtacatgtcactagagt	ttctgtaatttctcgggcag
29	ctgacatggagtttcctatt	ctcatactcagaattcctgc	ctcacagtcattaacggtca
30	aatatttcaccttctaaccc	aagccaggaaagtgttgcat	tctctgtgatggcagagatg
31	ggactgatgtagatagcatt	taaagtcttctggagggtgg	gtacgaggttcaggattcat
32	caatcacctctgtaccgtgt	cccgcaagttatgatgtatt	atctgctagtcactgtacag
33	gaagagatttaccatgtcct	acatcctcttgttcattagg	gactctagaaaacaccggct
34	ggccacagttataagacact	ttttagtgactcctgtttct	gaacatcagaggcagatggg
35	cctcattctatggactgttg	cttggctacagaaacccaat	ttactcaggagtcctaacga
36	ttccctaagaagtttagcac	cccaagatagtgctctatag	tgaagggacagtcagcaact
37	gcccaacacgttagagtata	aaggtcatggaacagagggg	tgaagggaagactccactca
38	caactcaagcacatggcttc	tcttggtaaattatgtgccc	tacaagtctctgcaggtcaa
39	ctgactgacaacgccatctc	agtcgggatgtggattacat	gttaagggatcagggttagg
40	cccattcaacgggataaaaa	ggactccaggaagtaggaat	tggaatggctccagagtaag
41		ggcaggtacgaatggaactt	agtggaagtgaaggcttctc
42		tccaggacaatgcaggtaat	ctcttcgtttccaaggaaga
43		tacagagaagaactggtccc	gcgtgaagacatctcacttt
44		agccattgtgcaaacttagg	gtctctgacagaacactgga
45		agtggaggacagtgtttagt	agcattgggcaaatagagca
46		catccagcttcgcaaagtaa	ctgggcagaacagctaagag
47		cttggtgcttgctaagtgaa	gagtgtcaagtgttaagggc
48		gaaaccatccatatcttgca	aaaacagcccaacccctttaa

Probe	Sox10	Pdgfra
1	tgaatcctcaccaccaaaca	taagagctggcaggagatga
2	gggagtgacgctgatggact	tctcgtttgggaggatagag
3	tcgtcctgaggaagtggaaa	gagtgtggttgtagtagcaa
4	acctctgataggtcttgttc	gactaaggaatcggtcatcc
5	cacacggggaacttgtcatc	agattcagttctgacgttgc
6	acaccatgaaggcgttcatg	agtgtacaccagtttgatgg
7	ttgtggaggtgagggtactg	ctgacttcgtgcaagttgac
8	tagtgtcttgctgagctcag	tctcggtgagattctctatc
9	ttgtcactttcgttcagcaa	atttgctctgataccttgtt
10	tctcagcctcctcaatgaag	atggtggtcatctacgagat
11	tttttgtgctgcatccggag	cagatcatccagtcgatttc
12	cgaggttggtacttgtagtc	agacattgctggccaaaact
13	ttgtagtgagcctgaatagc	aacgctgaggttgttctttg
14	tgtctttggggtggttggag	tgagttcagatcgcagagtg
15	acgttgccgaagtcgatgtg	caatcaccaacagcaccaac
16	atgttggacattacctcgtg	caaatgaccaccaggacgat
17	agctcagtcacatcaaaggt	aaatgcaccggatcccaaaa
18	atctgggaagtggacggctg	aaatgggacctgacttggtg
19	gcagactgagggaagtgtag	cccatagaagcagtattctg
20	aaactggggtcgtgagatgg	tgttggctcatgaagctatc
21	agggctgatggtcagaatag	caatccaaagatgtccaggt
22	tgcatggccataatagggtc	acatactgtgtggtatcagc
23	cagagatggcagtgtagagg	aaacctctttcctttcaagc
24	tgtgtgggactgtgggattg	cgtccgaaaggaggtttttg
25	ctaaggtcgggatagagtcg	cgtagttggaatcgtgcatg
26	tgggagagccaccaaacttc	acaccgatgtacgcattatc
27	tcatttctggggaagggtgg	caagtcctcttctcaacttt
28	tctcaggtcctgggatagag	ccggagaggagagttaacac
29	cttgctctccaaggagataa	tactcacggttggttagttt
30	aggcaatgacacagtagcac	tacttacactctgctctcta
31	gttcaaagctgtcctcagtg	cttcattcaacagcaccatt
32	ccaccctgctctttacaaag	caaatctctggggcaaaggt
33	ggcggagaaaggatcagagt	gactattcaagagcaccagg
34	tcccacattcaggtgaagag	aaatttcaaagttgccctcc
35	tgacacggaactggatctgg	tcaagggtaggaatagctgc
36	tcagttaggcaagggatgag	ggaagatgttcccaagtcaa
37	gagaagaaggctaggtggat	atggagctggttgcacaaag
38	gggcttgaggctgacagaag	cccatgtttcattcatacag
39	cgtgccatgctaactctgag	agattatgccagtaggtagt
40	acatggcaggggtttagtgt	aagggaaagttggggtttgc
41	gtgcaaggcaaaggtagact	tcagtcttcacacttactgt
42	taaggtatcgctggagcaaa	gccacgagtctagaaagacg
43	gagacagagctacgtacttt	gtatcatccaacttcatggt
44	tgggctgcagacaggagatg	attgaatctcagaggtgggt
45	cagcgatgtgttacatgtgg	cattttgtacaggttggcag
46	acatggcgactatggagata	tacgtccctcttcacaaata
47	ctggagtagctagagggata	gtcatacacattttggcagt
48	aggcaaccagaagcattgat	catcttgtattggaagaccc

Table S4. Related to Figure 7. qPCR primer sequences (5' -> 3')				
Gene	Accession No.	Sense	Antisense	Length (bp)
ALDH1A1	NM_000689	TGTTAGCTGATGCCGACTTG	CTGGCCCTGGTGGTAGAATA	71
CORIN	NM_006587	CATATCTCCATCGCCTCAGTTG	GGCAGGAGTCCATGACTGT	106
EN1	NM_001426	CGTGGCTTACTCCCCATTTA	TCTCGCTGTCTCTCCCTCTC	117
FOXA2	NM_021784	TTCAGGCCCGGCTAACTCT	AGTCTCGACCCCCACTTGCT	67
GAPDH	NM_002046	GAAGGTGAAGGTCGGAGTCA	TTGAGGTCAATGAAGGGGTC	117
LMX1A	NM_177398	GATCCCTTCCGACAGGGTCTC	GGTTTCCCACTCTGGACTGC	175
NANOG	NM_024865	ACAACTGGCCGAAGAATAGCA	GGTTCCCAGTCGGGTTCA	111
NR4A2	NM_006186	CAGCTCCGATTTCTTAACTCCAG	GGTGAGGTCCATGCTAAACTTGA	52
POU5F	NM_002701	AGGGCCCCATTTTGGTACC	TCAGTTTGAATGCATGGGAGAGC	136
SOX1	NM_005986	GCGGAGCTCGTCGCATT	GCGGTAACAACTACAAAAAACTTG	76
TH	NM_199292	ACTGGTTCACGGTGGAGTTC	TCTCAGGCTCCTCAGACAGG	117
